# A mitochondria-targeted H_2_O_2_/viscosity dual-responsive fluorescent probe for visualizing redox-biophysical remodeling in LPS-induced acute kidney injury

**DOI:** 10.1016/j.redox.2026.104294

**Published:** 2026-07-10

**Authors:** Junjie Wang, Rong Yu, Wei Chen, Xiaomin Ma, Xingzhou Peng, Fabiao Yu, Yongjun Zhu

**Affiliations:** a NHC Key Laboratory of Tropical Disease Control, School of Life Science and Medical Technology, Hainan Medical University, Haikou, 571199, China; b Department of Nephrology, Key Laboratory of Emergency and Trauma, Ministry of Education, Key Laboratory of Haikou Trauma, Key Laboratory of Hainan Trauma and Disaster Rescue, The First Affiliated Hospital, Hainan Medical University, Haikou, 571199, China; c School of Biomedical Engineering, Hainan University, Haikou, 570228, China

**Keywords:** Mitochondrial imaging, Redox-biophysical remodeling, H_2_O_2_, Viscosity, Acute kidney injury, Necroptosis-associated tubular injury

## Abstract

Renal tubular epithelial cells are highly susceptible to mitochondrial dysfunction during acute kidney injury (AKI), in which oxidative stress and microenvironmental remodeling occur before overt functional deterioration. Hydrogen peroxide (H_2_O_2_) is an important but nonspecific redox mediator, whereas mitochondrial viscosity provides a complementary biophysical readout associated with organelle stress, protein aggregation, membrane damage, and impaired molecular diffusion. Simultaneous imaging of mitochondrial H_2_O_2_ and viscosity may therefore provide a dual-parameter strategy for interrogating redox-biophysical remodeling during AKI-associated tubular injury. Here, we developed a mitochondria-targeted dual-responsive fluorescent probe, PB-PB-B(OH)2, that enables single-excitation dual-channel imaging of H_2_O_2_-associated oxidative stress and viscosity-related microenvironmental changes. PB-PB-B(OH)2 showed H_2_O_2_-responsive green emission and viscosity-sensitive red emission with limited channel cross-interference under the tested conditions. In HK-2 cells exposed to TNF-α or LPS, the probe visualized concurrent increases in mitochondrial oxidative stress-associated green fluorescence and viscosity-related red fluorescence, which were attenuated by NAC or Nec-1s treatment. In an LPS-induced AKI mouse model, PB-PB-B(OH)2 enabled dynamic renal imaging of injury-stage-dependent redox and viscosity changes, which correlated with histological injury, renal function markers, and necroptosis-related signaling proteins. Therapeutic intervention with NAC or Nec-1s reduced both fluorescence signals, supporting the use of this probe for imaging-based monitoring of renal injury progression and treatment response. These results establish PB-PB-B(OH)2 as a mitochondria-targeted dual-parameter molecular imaging tool for visualizing redox-biophysical remodeling in LPS-induced AKI, rather than as a clinically validated replacement for established AKI biomarkers.

## Highlights

• A mitochondria-targeted NIR two-photon probe enables H_2_O_2_/viscosity dual-channel imaging.
• Single-wavelength excitation visualizes necroptosis in LPS-induced AKI *in vivo.*
• TNF-α/LPS-injured HK-2 cells show coupled redox-biophysical remodeling.

## Graphical abstract

**Figure:**
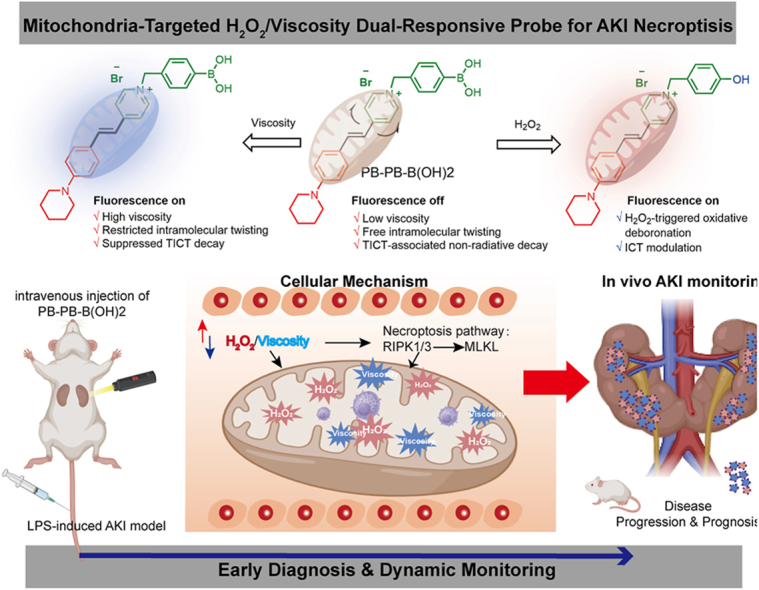

## 1 Introduction

Acute kidney injury (AKI) is a clinically heterogeneous syndrome characterized by a rapid decline in kidney function and is associated with high morbidity, mortality, and long-term risk of chronic kidney disease [1,2]. Despite decades of investigation, the early diagnosis and dynamic assessment of AKI remain a major clinical challenge. Current clinical criteria rely mainly on serum creatinine and urine output, which reflect functional deterioration rather than the initiating molecular injury. These indicators are often delayed, influenced by hemodynamic and metabolic factors, and insufficient to define the site, mechanism, and progression of tubular damage [[3], [4], [5]]. Although emerging biomarkers such as NGAL, KIM-1, and other damage- or stress-related molecules have improved risk stratification, they do not provide direct visualization of early organelle-level events in living renal tubular cells [6]. Therefore, imaging strategies capable of reporting molecular injury at the lesion site are needed to complement conventional functional readouts and provide early molecular-level information on AKI-associated tubular stress.

Renal tubular epithelial cells are the primary targets in AKI because of their high metabolic demand and dependence on mitochondrial oxidative metabolism [7]. Before overt renal dysfunction becomes clinically detectable, tubular cells undergo mitochondrial depolarization, respiratory chain disturbance, excessive reactive oxygen species generation, and activation of regulated cell death pathways [[8], [9], [10]]. Among these pathways, necroptosis has emerged as a major form of programmed necrosis involved in ischemic, septic, toxic, and inflammatory AKI [8,11]. Necroptosis is typically mediated by RIPK1/RIPK3/MLKL signaling and leads to plasma membrane rupture, release of damage-associated molecular patterns, and amplification of renal inflammation [12,13]. Importantly, mitochondrial redox imbalance may act as an upstream stress signal that facilitates necroptotic activation [8], thereby connecting early mitochondrial dysfunction with tubular cell rupture and subsequent inflammatory amplification. Thus, sensitive visualization of mitochondrial injury during necroptosis-associated tubular stress may provide an earlier molecular readout of AKI progression than late-stage functional indicators such as Cr and BUN in preclinical models.

Hydrogen peroxide (H_2_O_2_) is a central mitochondrial redox molecule because of its relative stability, diffusibility, and involvement in both physiological redox signaling and pathological oxidative injury [[14], [15], [16]]. Nevertheless, H_2_O_2_ is a broadly shared oxidative stress mediator rather than a disease- or death-mode-specific biomarker. Its elevation may occur in diverse pathological contexts [17,18], including inflammation, ischemia-reperfusion injury, drug-induced toxicity, and metabolic disorders, and therefore does not define AKI or necroptosis-associated tubular damage by itself. Mitochondrial viscosity provides a complementary biophysical indicator. As a parameter that affects molecular diffusion, enzyme activity, metabolite exchange, and local ROS propagation, viscosity is sensitive to mitochondrial swelling, membrane damage, protein aggregation, lipid peroxidation, and matrix microenvironmental remodeling [19,20]. Therefore, simultaneous imaging of mitochondrial H_2_O_2_ and viscosity offers a dual-dimensional strategy to distinguish isolated redox activation from redox stress accompanied by biophysical microenvironmental remodeling, thereby enabling a more integrated assessment of AKI-associated tubular injury.

Fluorescent probes provide sensitive, spatially resolved, and dynamic tools for monitoring intracellular redox biology in living systems. Given the importance of mitochondrial redox homeostasis and microenvironmental remodeling, numerous mitochondria-targeted probes have been developed for detecting ROS, reactive nitrogen species, pH, polarity, viscosity [[21], [22], [23], [24], [25], [26], [27]]. Recently, dual-responsive fluorescent probes have attracted increasing attention because they enable simultaneous visualization of multiple biochemical or biophysical parameters within specific organelles [[28], [29], [30], [31]]. Nevertheless, most reported ROS/viscosity dual-responsive systems have been developed primarily for probe construction or general oxidative stress imaging, and their integration with defined disease mechanisms remains limited (Table S1). Only a few mitochondrial H_2_O_2_/viscosity dual-responsive probes have been applied to neurological or ischemia-reperfusion-related models [20,28,32], whereas their use for visualizing redox-biophysical remodeling during LPS-induced AKI-associated tubular injury remains underexplored. This gap limits our ability to dynamically interrogate how mitochondrial oxidative stress and viscosity-related microenvironmental remodeling coexist during inflammatory renal tubular injury.

**Scheme 1:**
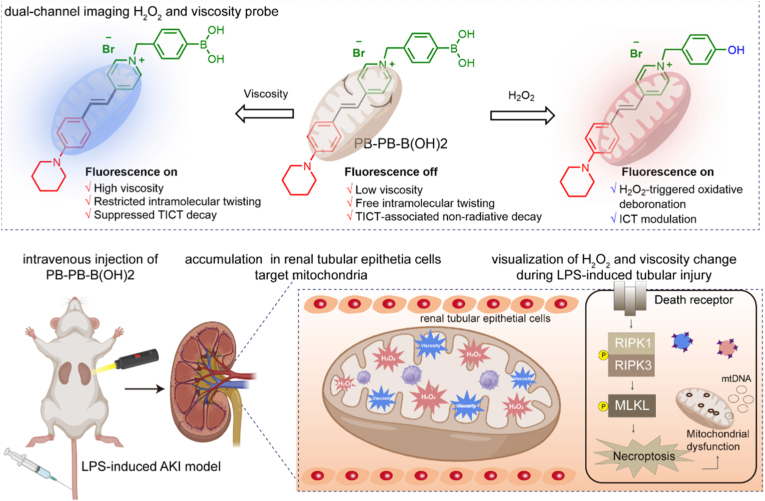
Molecular design and dual-response mechanism of PB-PB-B(OH)2 for mitochondrial H_2_O_2_/viscosity imaging, and schematic illustration of its application in visualizing redox–biophysical remodeling during LPS-induced AKI-associated tubular injury.

Herein, we report a mitochondria-targeted dual-responsive fluorescent probe, PB-PB-B(OH)2, for dual-parameter fluorescence imaging of mitochondrial H_2_O_2_-associated oxidative stress and viscosity-related microenvironmental remodeling during LPS-induced AKI (Scheme 1). The probe was constructed using a D-π-A hemicyanine/pyridinium scaffold, in which the cationic fluorophore favors mitochondrial accumulation, the phenylboronic acid unit enables H_2_O_2_-responsive activation, and the rotatable conjugated skeleton enables viscosity-sensitive fluorescence enhancement through restriction of intramolecular motion. This molecular design allows H_2_O_2_ and viscosity to be monitored as coupled redox-biophysical readouts rather than as isolated pathological markers. PB-PB-B(OH)2 was evaluated for optical response, selectivity, sensitivity, mitochondrial localization, and two-photon excitation capability. It was then applied to visualize mitochondrial H_2_O_2_/viscosity changes in renal tubular epithelial cells under inflammatory injury conditions and further used for imaging-based monitoring of LPS-induced AKI progression and therapeutic response. By connecting mitochondrial redox stress with viscosity-associated microenvironmental remodeling, this work provides a molecular imaging strategy for interrogating AKI-associated tubular injury.

## 2 Materials and methods

### 2.1 Materials, apparatus, and synthetic routes

See Supporting Information for details.

### 2.2 Cell culture and imaging

Cells were cultured in DMEM supplemented with 10% FBS in a humidified atmosphere of 5% CO_2_, and 95% air at 37 °C. Cells were seeded in glass-bottom cell culture dishes and allowed to adhere for 24 h. HK-2 cells were incubated with PB-PB-B(OH)2 (10 μM) at 37 °C for 30 min and then washed with PBS, followed by incubation with commercial probe MitoTracker Deep Red (100 nM), ERTracker Green (100 nM), LysoTracker Green (100 nM), BODIPY 493/503 (100 nM) for another 30 min. Cells were washed three times with PBS and then visualized by confocal microscopy.

### 2.3 Monitoring H_2_O_2_/viscosity changes using PB-PB-B(OH)2 probe in HK-2 cells

Cells were treated with H_2_O_2_ (0-80 μM) or nystatin (0-15 μM) for 30 min. The culture medium was then removed, and the cells were incubated with 10 μM PB-PB-B(OH)2 for 30 min. To inhibit ROS production, the cells were incubated with NAC (1 mM). Fluorescence imaging was performed using a confocal laser scanning microscope. For the green channel, emissions were collected from 480 to 580 nm with excitation of 405 nm; for the red channel, emissions were collected from 640 to 700 nm with excitation of 405 nm. The flow cytometry experiment was also conducted using a BD flow cytometer, and the data were analyzed with FlowJo software.

### 2.4 Monitoring of H_2_O_2_ accumulation and viscosity change in TNF-α-induced tubular cell injury

The HK-2 cells were treated with TNF-α (100 ng/mL) for 6, 12, and 24 h, the medium was removed, and the cells were incubated with 10 μM PB-PB-B(OH)2 for 30 min. To examine the contribution of ROS and RIPK1/necroptosis-related signaling, the cells were incubated with 100 ng/mL TNF-α in the presence of NAC (1 mM) or Nec-1s (1 μM). Fluorescence imaging was performed with confocal laser scanning microscope. The flow cytometry experiment was also conducted with BD flow cytometer, and the data were analyzed with FlowJo software.

### 2.5 Monitoring of H_2_O_2_ accumulation and viscosity change in LPS-induced tubular cell injury

The HK-2 cells were treated with LPS (20 μg/mL) for 12 or 24 h, then removed and finally incubated with 10 μM PB-PB-B(OH)2 for 30 min. To evaluate the involvement of ROS and RIPK1/necroptosis-related signaling, the cells were incubated with 20 μg/mL LPS in the presence of NAC (1 mM) or Nec-1s (1 μM). Fluorescence imaging was performed with confocal laser scanning microscope. The flow cytometry experiment was also conducted with BD flow cytometer, and the data were analyzed with FlowJo software.

### 2.6 *In vivo* imaging in LPS-induced AKI mouse models

All surgical procedures and experimental protocols were approved by the Animal Care and Use Committee of Hainan Medical University (HYLL-2024-103). 6-8-week-old male BALB/c mice of SPF grade were purchased from Guizhou Bio-Han Biotechnology Co., Ltd. (Guizhou, China). The AKI mouse model experiments were conducted as follows: the mice were randomly divided into three groups: control group, the LPS-treated group and the inhibitor-treated group. Then the mice in the control group received intraperitoneal saline (200 μL); the mice in the stimulated group were treated with LPS (10 mg/kg) by intraperitoneal injection for 12, 24 and 48 h, followed by intravenous injection of PB-PB-B(OH)2 (250 μM, 200 μL); the mice in the inhibited group were successively treated with NAC (400 mg/kg) or Nec-1s (1.5 mg/kg) for 30 min, followed by intraperitoneal (*i.p.*) injection of LPS (10 mg/kg) for 24 h, then the PB-PB-B(OH)2 (250 μM, 200 μL) was injected intravenously (*i.v.*). Following anesthesia, *in vivo* fluorescence imaging was performed using a PerkinElmer IVIS Lumina XRMS small animal optical imaging system. The imaging parameters were set with an excitation wavelength of 405 nm, and fluorescence emission was recorded at 560 nm and 660 nm. After imaging, the mice were euthanized, and the kidneys were excised for *ex vivo* fluorescence imaging.

### 2.7 Apoptosis/necrosis assay

An FITC-Annexin V/propidium iodide (PI) apoptosis detection kit (Shanghai Yesen Biotechnology Co. Ltd) was used to quantify necrotic and apoptotic cell populations of HK-2 cells induced by TNF-α or LPS. Western blotting was performed to detect the expression of necroptosis-related proteins RIPK1, RIPK3 and MLKL. The assay was performed according to the manufacturer's protocol.

### 2.8 Statistical analysis

Statistical analysis was performed using GraphPad Prism (GraphPad Software, Inc., La Jolla, CA, USA). For a two-group comparison, a Student's t-test was performed for the statistical analysis. For multiple comparisons, the data were analyzed using one-way ANOVA with Tukey's multiple comparison test. Data were presented as mean ± SD. Significant differences between the groups were labeled as n.s. for non-significant differences, or * for *p* < 0.05, ** for *p* < 0.01, *** for *p* < 0.001 and **** for *p* < 0.0001.

## 3 Results and discussion

### 3.1 Design and synthesis of PB-PB-B(OH)2

**Scheme 2:**
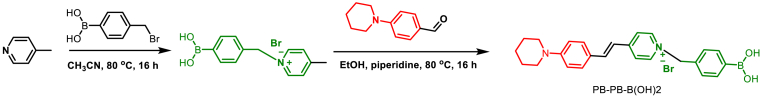
The synthetic route to PB-PB-B(OH)2.

We first designed PB-PB-B(OH)2 using an asymmetric strategy (Scheme 2). D-π-A hemicyanine/pyridinium-based fluorescent probes are widely used in bioimaging owing to their excellent optical properties and favorable biocompatibility, and their emission properties are largely determined by the intramolecular charge transfer (ICT) effect between donors and acceptors. By adjusting the electron-donating and electron-withdrawing moieties at both ends of the vinyl group, this D-π-A structure enhances ICT interactions, while also allowing viscosity-dependent restriction of twisted intramolecular charge transfer (TICT)-associated non-radiative decay [33] and potential two-photon absorption effects [34]. The former enables strong fluorescence emission in response to viscosity differences caused by pathological conditions, while the latter provides a basis for enhanced light penetration. Phenylboronic acid is utilized as a reaction site for H_2_O_2_ sensing, and H_2_O_2_-triggered oxidative deboronation of the phenylboronic acid group induces changes in the probe's electronic structure and fluorescence by modulating the ICT process. A pyridinium cation, serving as a linker reaction site and electron-accepting unit, provides mitochondrial targeting, improves aqueous compatibility, and enhances biocompatibility. Additionally, the synthetic route of PB-PB-B(OH)2 was simple and straightforward, including nucleophilic substitution reactions and Knoevenagel reactions. The characterization data and experimental methods of this probe were described in detail in the Supporting Information (Figs. S1–4).

### 3.2 Photophysical properties and fluorescence response of PB-PB-B(OH)2 probe to H_2_O_2_/viscosity

First, the photophysical properties of PB-PB-B(OH)2 was investigated. The solvatochromic behavior of PB-PB-B(OH)2 was systematically investigated in various organic solvents. Fluorescence emission analysis corroborated this ICT-based mechanism (Fig. S5), showing a general decrease in intensity with increasing solvent polarity from 1,4-dioxane to PBS. As shown in Table S2, PB-PB-B(OH)2 exhibited maximum absorption and emission peaks at 410 nm and 547 nm in PBS buffer, respectively. The fluorescence quantum yield ($\phi_{FL}$) of PB-PB-B(OH)2 was determined to be 0.19 ± 0.015 in the same buffer. The two-photon absorption cross-sections (*δ*) were further measured under excitation at wavelengths ranging from 800 to 1000 nm, with a maximum *δ* value of 71.6 GM at 850 nm (Fig. S6).

**Fig. 1:**
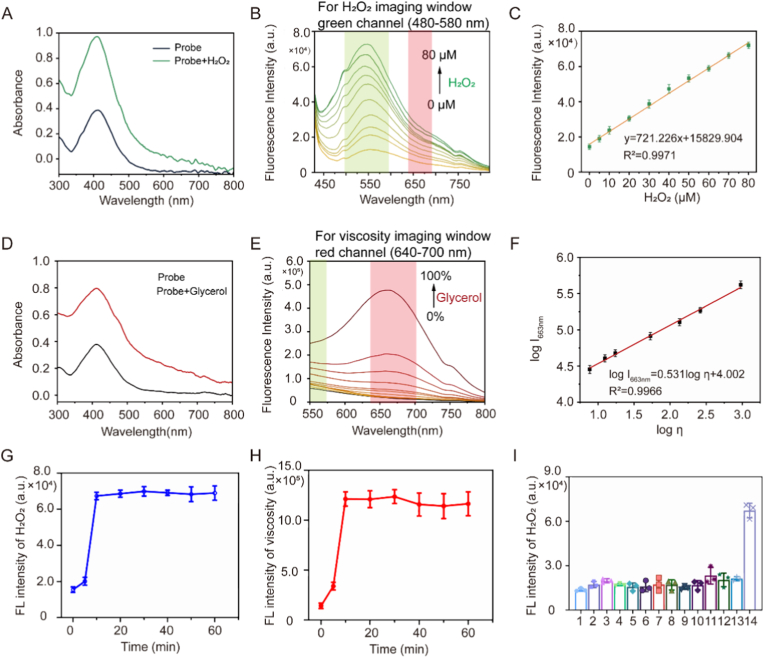
Spectral responses of the PB-PB-B(OH)2 toward H_2_O_2_ and viscosity under various conditions. (A) UV-vis absorption spectra of the probe (10 μM) in the absence or presence of H_2_O_2_ (80 μM) in PBS. (B) Fluorescence spectra of the probe (10 μM) upon incubation with various concentrations of H_2_O_2_ (0-80 μM) in PBS. (C) Linear relationship between fluorescence intensity and H_2_O_2_ concentrations. $\lambda_{ex}$ = 410 nm, $\lambda_{em}$ = 547 nm. (D) UV-vis absorption spectra of the probe (10 μM) in the absence and presence of glycerol (100%). (E) Fluorescence spectra of the probe (10 μM) in glycerol-PBS mixtures with different viscosities. (F) Linear relationship of fluorescence intensity (I_663_) and viscosity (η) described with Förster-Hoffmann equation. $\lambda_{ex}$ = 410 nm, $\lambda_{em}$ = 663 nm. (G) Fluorescence intensity at 547 nm of the probe after being exposed to H_2_O_2_ (80 μM) for different incubation times in PBS. (H) Fluorescence intensities at 663 nm of the probe after being exposed to viscosity (glycerol: PBS = 8:2) for different incubation times in PBS. (I) Fluorescence intensity at 547 nm in the presence of different biological species. 1: PBS; 2: GSH; 3: Hcy; 4: Cys; 5: NAC; 6: Nec-1s; 7: Na^+^; 8: Fe^3+^; 9: Ca^2+^; 10: •OH; 11: OCl^−^; 12: O_2_•^-^; 13: ONOO^−^; 14: H_2_O_2._

Next, the dual-response of PB-PB-B(OH)2 toward H_2_O_2_ and viscosity was studied. UV-vis absorption spectra showed a significant absorbance enhancement upon H_2_O_2_ addition (Fig. 1A), suggesting the oxidative activation of the probe. As illustrated in Fig. 1B, the fluorescence emission at 547 nm remained negligible in the absence of H_2_O_2_, but underwent significant amplification upon H_2_O_2_ titration, suggesting H_2_O_2_-responsive behavior. A linear correlation between the fluorescence intensities (at 547 nm) and H_2_O_2_ concentrations in the range of 0-80 μM was obtained (Fig. 1C). The limit of detection (LOD) and limit of quantification (LOQ) of the probe for H_2_O_2_ were 0.49 μM (3 σ/*k*) and 1.65 μM (10 σ/*k*), respectively. These results indicated that PB-PB-B(OH)2 has high sensitivity to H_2_O_2_ due to the H_2_O_2_-responsive phenylboronic acid cleavage. To evaluate the viscosity-responsive characteristics of PB-PB-B(OH)2, a series of glycerol-PBS mixtures with varying compositions were prepared (Table S3). As presented in Fig. 1E, the fluorescence intensity increased dramatically with rising viscosity, and an approximately 26.5-fold fluorescence enhancement at 663 nm was achieved in 100% glycerol (Fig. S7). These results collectively demonstrated that PB-PB-B(OH)2 is sensitive to viscosity, which may be attributed to the restriction of intramolecular rotation by increased viscosity. The relationship between fluorescence intensity and the medium viscosity (η) was analyzed by the Förster-Hoffmann equation (log F = κ log η +C, with κ denoting the dye-dependent constant and C representing the concentration and temperature-dependent offset; Fig. 1F). The Förster–Hoffmann analysis showed a good linear relationship between log F and log η (R^2^ = 0.99) over the tested glycerol/PBS viscosity range (0.99 - 953 cP), supporting the viscosity-responsive behavior of PB-PB-B(OH)2. These calibration experiments provide a physicochemical basis for using the red channel to report relative viscosity changes in biological imaging, rather than absolute intracellular viscosity values.

To further evaluate potential channel crosstalk, *in vitro* cross-interference experiments were analyzed by separately adjusting H_2_O_2_ concentration and medium viscosity under defined *in vitro* conditions. These *in vitro* cross-interference experiments showed spectrally distinguishable dual-response behavior with limited cross-interference under the tested conditions. Increasing H_2_O_2_ concentrations predominantly enhanced the emission band corresponding to the green fluorescence window (480-580 nm), whereas no obvious additional activation of the red-emissive region was observed under the tested *in vitro* conditions (Fig. 1B). Conversely, when viscosity was increased by changing the glycerol fraction, the fluorescence enhancement mainly occurred in the red-emissive region, while the green-emissive region showed only minor changes under the glycerol/PBS conditions (Fig. 1E). Considering that H_2_O_2_ and viscosity coexist in living systems, additional experiments were performed under controlled H_2_O_2_ and viscosity conditions (Fig. S8). At the H_2_O_2_-responsive emission wavelength of 547 nm, the fluorescence intensity increased progressively with H_2_O_2_ concentration in both PBS and 80% glycerol/PBS. The absolute fluorescence intensity was higher in 80% glycerol/PBS than in PBS, indicating that viscosity can modulate the basal fluorescence intensity of the green-channel readout. Nevertheless, the concentration-dependent H_2_O_2_ response was retained under both conditions. Conversely, the Förster–Hoffmann plots of log F versus log η in the absence and presence of H_2_O_2_ were nearly overlapped, indicating that H_2_O_2_ had limited influence on viscosity detection. These results indicated that H_2_O_2_-responsive phenylboronic acid cleavage and the viscosity-responsive restriction of intramolecular rotation are spectrally distinguishable and exhibit limited cross-interference under the tested conditions, rather than complete independence in all biological contexts.

In addition, the temporal characterization of the fluorescence response towards H_2_O_2_ and viscosity of PB-PB-B(OH)2 were presented in Fig. 1G and H, respectively. Fluorescence intensification proceeded rapidly during the initial incubation phase, with saturation achieved within approximately 15 min. The expeditious response kinetics suggested that PB-PB-B(OH)2 has considerable potential for application as a fluorescent probe in biological imaging systems. To assess selectivity, PB-PB-B(OH)2 was exposed to representative inorganic ions, endogenous biomolecules, and other potential interfering agents, and the fluorescence intensities were recorded as displayed in Fig. 1I and S9. The various substances, including several kinds of reactive oxygen species (ONOO^−^, ClO^−^, *etc*), reactive sulfur species (GSH, Hcy, Cys, NAC), inorganic anions (Cl-, CO_3_^2−^, HPO_4_^2−^, *etc*) and metal cations (Cu^2+^, Fe^2+^, *etc*) showed negligible fluorescence changes relative to the PBS group. By contrast, the probe exhibited significant fluorescence enhancement after treatment with H_2_O_2_. These findings established that PB-PB-B(OH)2 possesses high selectivity for H_2_O_2_ even in the presence of other substances, supporting its application in complex biological environments. Furthermore, the fluorescence intensity of PB-PB-B(OH)2 exhibited a certain pH dependence (Fig. S10). Notably, within the physiologically relevant pH window (pH 6-8), the fluorescence intensity remained largely unchanged, indicating that the probe response is minimally affected by pH fluctuations under biological imaging conditions. In contrast, pronounced fluorescence changes were observed only under strongly acidic (pH 4) or alkaline environments (pH 9). These results indicated that PB-PB-B(OH)2 remains applicable for AKI-related biological imaging under physiological conditions.

### 3.3 Fluorescence imaging of mitochondrial H_2_O_2_ and viscosity in living cells

**Fig. 2:**
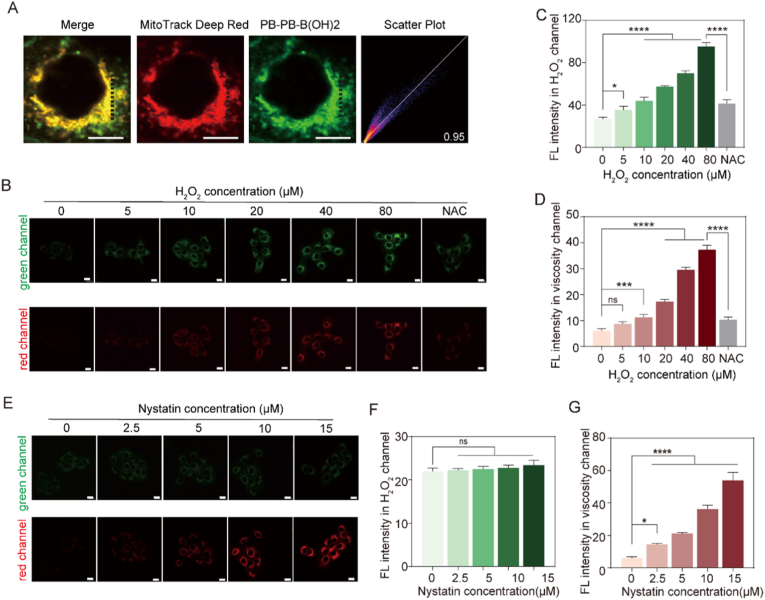
Fluorescence imaging of mitochondrial H_2_O_2_ and viscosity changes using PB-PB-B(OH)2 probe in HK-2 cells. (A) Mitochondrial multicolor imaging of MitoTracker Deep Red (red fluorescence) and PB-PB-B(OH)2 (green fluorescence) in HK-2 cells. Co-localization areas of green and red channels. Scale bar: 10 μm. (B) Fluorescence images of mitochondrial H_2_O_2_ change in HK-2 cells under different concentrations of H_2_O_2_ (0-80 μM) or NAC (1 mM) for 30 min. Green channel for H_2_O_2_: $\lambda_{ex}$ = 405 nm, $\lambda_{em}$ = 480-580 nm; red channel for viscosity: $\lambda_{ex}$ = 405 nm, $\lambda_{em}$ = 640-700 nm. (C-D) Quantification of fluorescence intensities of the fluorescence images in Fig. 2B. (E) Fluorescence images of mitochondrial viscosity change in HK-2 cells under different concentrations of nystatin (0-15 μM) for 30 min. Green channel for H_2_O_2_: $\lambda_{ex}$ = 405 nm, $\lambda_{em}$ = 480-580 nm; red channel for viscosity: $\lambda_{ex}$ = 405 nm, $\lambda_{em}$ = 640-700 nm. (F-G) Quantification of fluorescence intensities of the fluorescence images in Fig. 2E. Data are represented as mean ± SD (n = 3); Significant differences were analyzed by one-way ANOVA with Tukey's multiple comparison test. Pairwise comparisons are indicated by horizontal brackets in the graphs. n.s. (no significant difference); *P < 0.05; **P < 0.01; ***P < 0.001; ****P < 0.0001.

The CCK-8 assay showed that PB-PB-B(OH)2 had low cytotoxicity (Fig. S11). A good mitochondrial targeting of PB-PB-B(OH)2 was confirmed by the colocalization experiments with MitoTracker Deep Red (Fig. 2A), which showed a high Pearson's correlation coefficient (PCC) 0.95 and substantial spatial overlap (Fig. S12). When colocalized with other commercial dyes, PB-PB-B(OH)2 showed weak colocalization with ERTracker Green (PCC = 0.21) and moderate partial overlap with LysoTracker Green and BODIPY (PCC = 0.65 and 0.68, respectively), indicating preferential mitochondrial accumulation (Fig. S13). Next, the performance of PB-PB-B(OH)2 in imaging mitochondrial H_2_O_2_ and viscosity changes were evaluated. Exogenous H_2_O_2_ was first used as a model oxidative stimulus to validate the cellular responsiveness of PB-PB-B(OH)2, rather than as direct evidence for endogenous H_2_O_2_ monitoring. As shown in Fig. 2B–D, the fluorescence intensity of the green channel (H_2_O_2_-responsive channel, 480-580 nm) increased progressively with increasing H_2_O_2_ concentration. Quantitative analysis showed that the green-channel signal increased by approximately 1.3-, 1.7-, 2.2-, 2.6-, and 3.6-fold after treatment with 5, 10, 20, 40, and 80 μM H_2_O_2_, respectively. These results confirmed intracellular H_2_O_2_-responsive activation of the probe in living cells. Notably, the red channel (viscosity-responsive channel, 640-700 nm) also exhibited an accompanying increase under H_2_O_2_ stimulation, reaching approximately 1.4-, 1.9-, 2.9-, 4.9-, and 6.1-fold at the corresponding H_2_O_2_ concentrations. In the presence of NAC (ROS inhibitor), both the green and red fluorescence responses were markedly suppressed, decreasing to approximately 1.6- and 1.7-fold of the basal level. The results of the flow cytometry analysis were consistent with the fluorescence imaging results (Fig. S14). The accompanying increase in the red-channel signal should not be interpreted as direct chemical activation of the viscosity channel by H_2_O_2_. Instead, it likely reflects oxidative stress-induced mitochondrial dysfunction and viscosity-related microenvironmental alterations. Therefore, the dual-channel increase observed in living cells indicates biologically coupled oxidative and biophysical changes rather than direct spectral crosstalk between the two channels.

Since nystatin has been reported to cause an increase in mitochondrial viscosity, the viscosity response of probe was investigated. As shown in Fig. 2E, nystatin treatment induced a concentration-dependent enhancement of the red channel fluorescence intensity (viscosity-responsive channel, 640-700 nm), whereas the green channel fluorescence intensity (H_2_O_2_-responsive channel, 480-580 nm) remained unchanged. Quantitative analysis showed that the red-channel signal increased by approximately 2.4-, 3.5-, 6.0-, and 8.9-fold after treatment with 2.5, 5, 10, and 15 μM nystatin, respectively (Fig. 2F). In contrast, the green-channel signal showed only minimal fluctuation within approximately 1.1-fold over the same nystatin concentration range, with no statistically significant difference (Fig. 2G). A similar trend was observed in the flow cytometry analysis in Fig. S15. These results indicated that viscosity-related intracellular microenvironmental changes predominantly activated the red channel without significantly triggering the H_2_O_2_-responsive green signal. Therefore, these data suggested limited influence of viscosity-related changes on the green channel readout under the tested cellular conditions.

### 3.4 Mitochondrial H_2_O_2_/viscosity changes under TNF-α-induced tubular cell injury

**Fig. 3:**
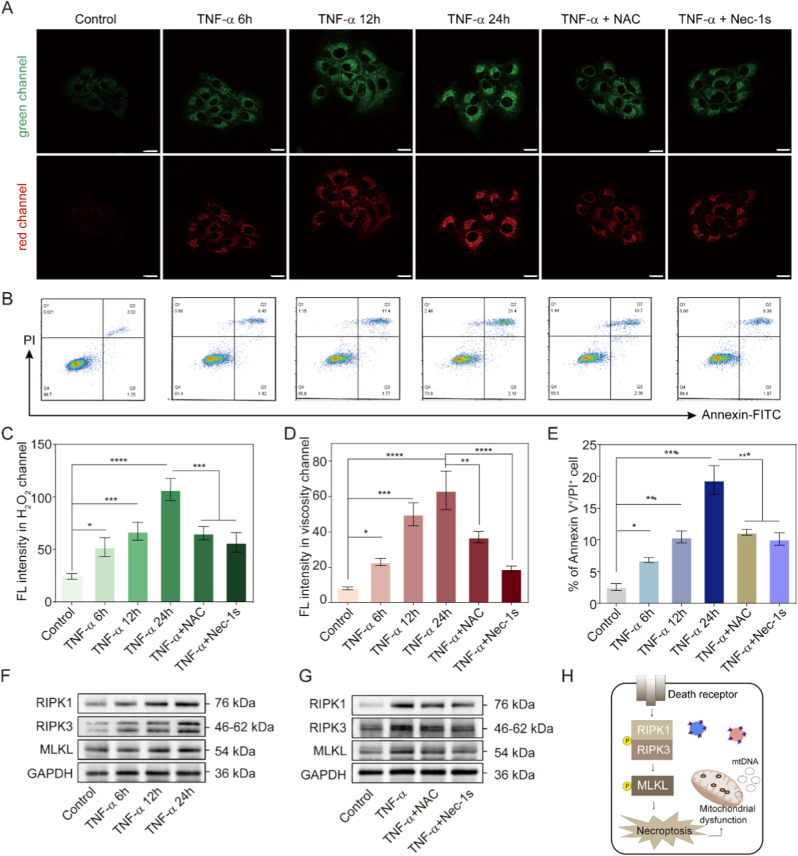
Monitoring of H_2_O_2_ accumulation and viscosity change in TNF-α-induced tubular cell injury. (A) Fluorescence images of HK-2 cells treated with TNF-α (100 ng/mL) for 6, 12, or 24 h, with or without NAC (1 mM) or Nec-1s (1 μM). Green channel for H_2_O_2_: $\lambda_{ex}$ = 405 nm, $\lambda_{em}$ = 480-580 nm; red channel for viscosity: $\lambda_{ex}$ = 405 nm, $\lambda_{em}$ = 640-700 nm. Scale bar: 10 μm. (B) Apoptosis/necrosis analysis of Annexin V-FITC/PI analyzed by flow cytometry. (C-D) Relative pixel intensity of the fluorescence images in (A). (E) Quantification of necrotic/apoptotic cells. (F) Western blotting analysis of necroptosis-related proteins RIPK1, RIPK3, and MLKL after TNF-α stimulation for 6, 12, and 24 h. (G) Western blotting analysis of necroptosis-related proteins RIPK1, RIPK3, and MLKL with or without NAC and Nec-1s. (H) Schematic illustration of TNF-α-induced mitochondrial redox-biophysical remodeling and necroptosis-related signaling. Data are represented as mean ± SD (n = 3); Significant differences were analyzed by one-way ANOVA with Tukey's multiple comparison test. The compared groups are indicated by horizontal brackets in the graphs. n.s. (no significant difference); *P < 0.05; **P < 0.01; ***P < 0.001; ****P < 0.0001.

To assess whether PB-PB-B(OH)2 could report endogenously induced oxidative stress-associated changes, TNF-α was used as an inflammatory stimulus. Unlike exogenous H_2_O_2_, TNF-α does not directly supply H_2_O_2_ but induces intracellular ROS/H_2_O_2_-associated oxidative stress through inflammatory signaling. The HK-2 cells were incubated with TNF-α (100 ng/mL) for 6 h, 12 h, 24 h, and then treated with the ROS inhibitor NAC (1 mM) or necrosis small molecule inhibitor Nec-1s (1 μM). As shown in Fig. 3A, both green and red fluorescence signals increased in a time-dependent manner after TNF-α treatment, suggesting concurrent mitochondrial oxidative stress and viscosity-related microenvironmental remodeling. NAC attenuated the green-channel response, supporting its association with ROS/H_2_O_2_-related oxidative stress. Nec-1s also reduced the dual-channel fluorescence changes, suggesting that these redox–biophysical alterations are associated with regulated necrotic cell injury rather than being nonspecific fluorescence fluctuations. The corresponding quantification results also confirmed this trend (Fig. 3C and D).

Cell-death analysis further supported the injury-associated interpretation of the fluorescence signals. As shown in Fig. 3B and E, the Annexin V/PI analysis showed that the Annexin V^+^/PI^+^ cell population increased from approximately 5% to 20% after TNF-α stimulation and was partially reduced by NAC or Nec-1s treatment. After treating HK-2 cells with TNF-α for 24 h, the expression levels of necroptosis-related proteins [35,36], including RIPK1, RIPK3, and MLKL, were increased (Fig. 3F and G). These changes were partially reversed when the cells were pre-incubated with NAC or Nec-1s (Fig. S16). Together, these results support an association between TNF-α-induced mitochondrial redox–biophysical remodeling and necroptosis-related tubular cell injury. Therefore, PB-PB-B(OH)2 can be used as a dual-parameter imaging tool to monitor H_2_O_2_-associated oxidative stress and viscosity-related microenvironmental changes during TNF-α-induced tubular injury (Fig. 3H).

### 3.5 Visualization of mitochondrial H_2_O_2_ accumulation and viscosity remodeling in LPS-induced tubular cell injury

**Fig. 4:**
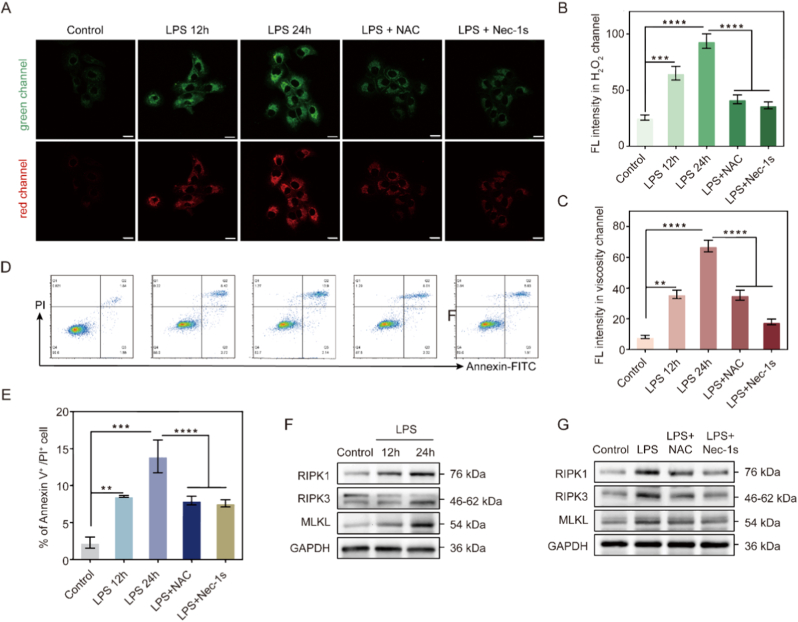
Visualization of mitochondrial H_2_O_2_ accumulation and viscosity remodeling in LPS-induced tubular cell injury. (A) Fluorescence images of HK-2 cells treated with LPS (20 μg/mL) for 12 or 24 h, co-treated with NAC (1 mM) or Nec-1s (1 μM). Green channel for H_2_O_2_: $\lambda_{ex}$ = 405 nm, $\lambda_{em}$ = 480-580 nm; red channel for viscosity: $\lambda_{ex}$ = 405 nm, $\lambda_{em}$ = 640-700 nm. Scale bar: 10 μm. (B–C) Relative pixel intensity of the fluorescence images in (A). (D) Apoptosis/necrosis assays of Annexin V-FITC/PI tested by flow cytometry. (E) The histograms of necrotic/apoptotic data in (D). (F) Western blotting analysis of necroptosis-related proteins RIPK1, RIPK3, and MLKL after LPS stimulation for 12 and 24 h. (G) Western blotting analysis of RIPK1, RIPK3, and MLKL after 24 h of LPS stimulation with or without NAC or Nec-1s treatment. Data are represented as mean ± SD (n = 3); Significant differences were analyzed by one-way ANOVA with Tukey's multiple comparison test. The compared groups are indicated by horizontal brackets in the graphs. n.s. (no significant difference); *P < 0.05; **P < 0.01; ***P < 0.001; ****P < 0.0001.

Since lipopolysaccharide (LPS) is a key mediator of AKI and can induce oxidative stress, mitochondrial dysfunction, and regulated necrotic signaling in renal tubular epithelial cells, LPS-induced tubular injury may be accompanied by changes in mitochondrial H_2_O_2_ levels and viscosity-related microenvironmental remodeling. Thus, visualization of mitochondrial H_2_O_2_ and viscosity changes may provide a redox-biophysical readout of tubular injury progression. As shown in Fig. 4A, the fluorescence signals of PB-PB-B(OH)2 in the green channel and the red channel were significantly increased after LPS stimulation for 12 h and 24 h, and reached their maximum at 24 h. Interestingly, the fluorescence signals of H_2_O_2_ and viscosity were down-regulated after treatment with NAC or Nec-1s in AKI models. The corresponding quantification data were in line with the above trends (Fig. 4B and C). These results suggest that mitochondrial H_2_O_2_ accumulation and viscosity elevation are closely associated with LPS-induced tubular cell injury. To further assess LPS-induced HK-2 cell injury and its association with mitochondrial redox–biophysical changes, cell death was evaluated by Annexin V/PI flow cytometry (Fig. 4D). Annexin V/PI analysis showed increased PI-positive/late-stage cell death after 24 h of LPS stimulation, whereas this increase was reduced in the NAC- and Nec-1s-treated groups (Fig. 4E). Consistently, Western blot analysis revealed that LPS increased the expression of necroptosis-related proteins, including RIPK1, RIPK3, and MLKL, whereas NAC or Nec-1s partially reversed these changes (Fig. 4F and G and S17). Therefore, PB-PB-B(OH)2 enables visualization of mitochondrial redox-biophysical remodeling associated with LPS-induced tubular cell injury, which is accompanied by changes in necroptosis-related signaling proteins. It should be noted that PB-PB-B(OH)2 is not a necroptosis-specific fluorescent reporter. The green and red fluorescence signals reflect mitochondrial H_2_O_2_-associated oxidative stress and viscosity-related microenvironmental remodeling, respectively. In the TNF-α- and LPS-induced injury models, these redox-biophysical changes were accompanied by increased RIPK1/RIPK3/MLKL signaling and were partially attenuated by Nec-1s treatment, supporting their association with necroptosis-related tubular injury. However, because mitochondrial oxidative stress and viscosity remodeling may also occur in other forms of cellular injury, the fluorescence signals should be interpreted as injury-associated redox-biophysical readouts rather than specific indicators of necroptosis.

### 3.6 *In vivo* fluorescence imaging of renal H_2_O_2_-associated and viscosity-associated changes in LPS-induced AKI mouse models

Before evaluating the applicability of the PB-PB-B(OH)2 for detecting H_2_O_2_ and viscosity *in vivo*, the biosafety of the PB-PB-B(OH)2 was investigated. The BALB/c mice were injected with the probe (250 μM, 200 μL) *via* the tail vein as the probe-treated group. The mice were injected with the same volume of saline as the control group. Assays including the H&E staining of major organs, complete blood counts, and liver and kidney function tests were performed 24 h post-injection. H&E analysis revealed no obvious damage to the major organs in either the probe-treated group or control group (Fig. S18A). Concurrently, complete blood counts, liver and renal function indicators remained within normal physiological ranges in probe-treated groups (Fig. S18). These results verified the favorable biocompatibility of PB-PB-B(OH)2 and supported its application *in vivo* investigations.

**Fig. 5:**
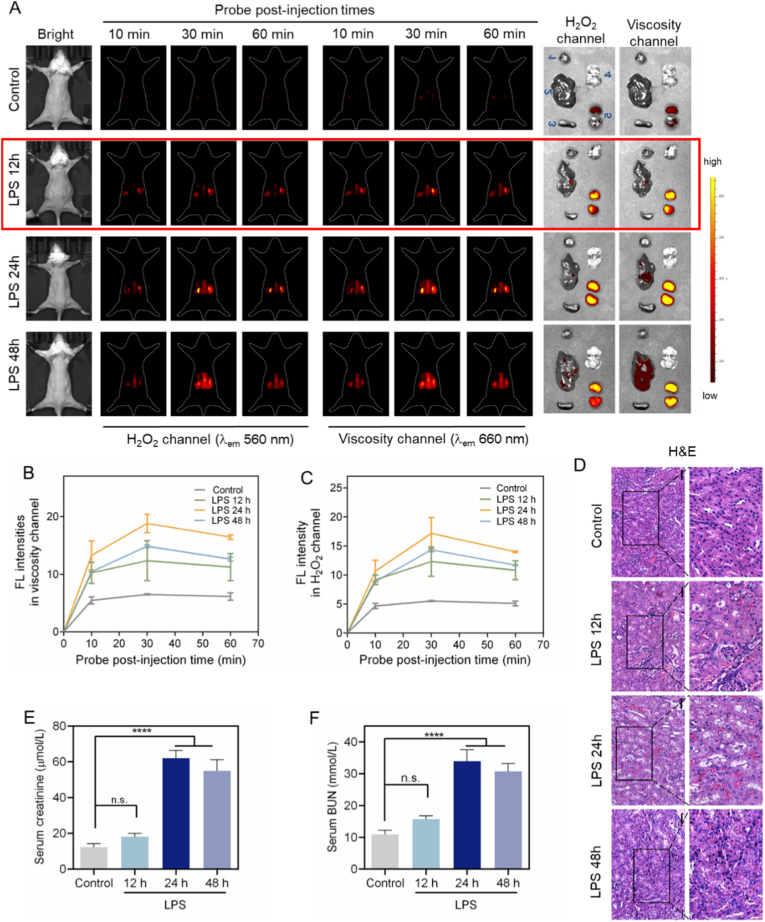
Real-time *in vivo* dual-channel imaging of LPS-induced AKI. (A) Fluorescence images of living mice with *i.v.* injection of PB-PB-B(OH)2 after treatment with LPS (10 mg/kg) for 12, 24 or 48 h. *Ex vivo* imaging of major organs in AKI mice after intravenous injection of PB-PB-B(OH)2 for 60 min (for H_2_O_2_: $\lambda_{em}$ = 560 nm; for viscosity: $\lambda_{em}$ = 660 nm). (B–C) The dynamic fluorescence intensity of kidneys as a function of time post-injection of probe in living mice after treatment with LPS for 12, 24 or 48 h, respectively. (D) Representative H&E staining of kidney tissue sections from mice treated with LPS for 12, 24 or 48 h. Scale bar: 50 μm. (E-F) Quantification of renal injury severity *via* serum creatinine (E), and BUN (F). Data are shown as mean ± SD (n = 5); statistical significance was determined by one-way ANOVA with Tukey's multiple comparison test. The compared groups are indicated by horizontal brackets in the graphs. n.s. (no significant difference); *P < 0.05; **P < 0.01; ***P < 0.001; ****P < 0.0001.

Next, the LPS-induced AKI mouse model was established to examine whether the dual-channel probe could visualize H_2_O_2_-associated and viscosity-associated fluorescence changes during AKI progression. Following intravenous administration of the probe (PB-PB-B(OH)2, 250 μM, 200 μL), *in vivo* imaging was performed at 12, 24 and 48 h post-LPS treatment. Fluorescence signals in both the H_2_O_2_ channel ($\lambda_{em}$ = 560 nm) and viscosity channel ($\lambda_{em}$ = 660 nm) showed time-dependent fluorescence patterns in the kidney, with maximal signal detected approximately 30 min post-injection of probe and then declining gradually thereafter (Fig. 5A). At 12 h post-treatment with LPS, quantitative analysis revealed 2.2-fold and 1.9-fold increases in H_2_O_2_- and viscosity-channel fluorescence relative to the control, respectively. The temporal profile of signal evolution remained qualitatively consistent across the 24 and 48 h post-treatment with LPS (Fig. 5B and C). Peak fluorescence intensity was recorded at 24 h post-LPS administration, representing 1.4-fold and 1.2-fold enhancement relative to the 12 h and 48 h measurements, respectively. However, fluorescence intensity decreased at 48 h post-treatment with LPS but remained above the control level. This phenomenon may be partly associated with attenuation of the acute LPS-induced inflammatory insult over time, possibly due to gradual clearance of LPS [37]. In addition, previous studies have shown that renal dysfunction in endotoxemia-associated AKI can partially recover after the peak injury phase [38]. The *ex vivo* imaging of major organs showed prominent renal fluorescence signals under the tested conditions. In addition, the kidney section imaging revealed that fluorescence signals were retained in renal tubular epithelial cells (Fig. S19). Meanwhile, two-photon fluorescence imaging was performed in tissue sections and compared with one-photon imaging. As shown in Fig. S20, two-photon excited fluorescence reached an imaging depth of 74 μm (z-stack of 16 slices and a step size of 5.0 μm) while one-photon excited fluorescence imaging only had a depth of 20 μm (z-stack of 6 slices and a step size of 5.0 μm). These results indicate that the two-photon fluorescence mode is superior to the one-photon mode in deep-tissue imaging.

Because LPS challenge can induce systemic inflammatory responses, the observed fluorescence changes should not be interpreted as evidence that H_2_O_2_-associated oxidative stress and viscosity-related remodeling occur exclusively in the kidney. These imaging readouts were further interpreted together with renal histopathology, serum Cr/BUN levels, and necroptosis-related protein expression to support their association with LPS-induced AKI progression. The H&E section results (Fig. 5D) showed that LPS treatment caused typical inflammatory damage such as tubular swelling, tubular deformation, and interstitial inflammatory cell infiltration. The degree of damage was most severe at 24 h post-drug treatment, which was highly consistent with the imaging results. Furthermore, the expression levels of key necroptosis proteins (RIPK1, RIPK3, and MLKL) in renal tissue were significantly upregulated at 24 h after LPS treatment, showing a positive correlation with the changes in fluorescence intensity (Fig. S21). These results indicate that LPS-induced AKI is accompanied by upregulation of necroptosis-related signaling proteins, and that the PB-PB-B(OH)2 fluorescence signals are positively associated with renal histological injury and necroptosis-related molecular changes. Commercial assays were used to measure serum Cr and BUN in blood (Fig. 5E and F). Serum Cr and BUN levels increased significantly by 5.0- and 3.1-fold at 24 h and by 4.4- and 2.8-fold at 48 h, respectively, after LPS treatment. Compared with serum Cr and BUN, which showed significant elevation mainly at later time points in this model, PB-PB-B(OH)2 detected renal fluorescence changes as early as 12 h after LPS challenge. These findings suggest that PB-PB-B(OH)2 provides an early molecular imaging readout of AKI-associated renal stress in the LPS-induced mouse model, rather than a clinically validated diagnostic replacement for established AKI biomarkers.

### 3.7 Therapeutic evaluation of LPS-induced AKI *in vivo* imaging

**Fig. 6:**
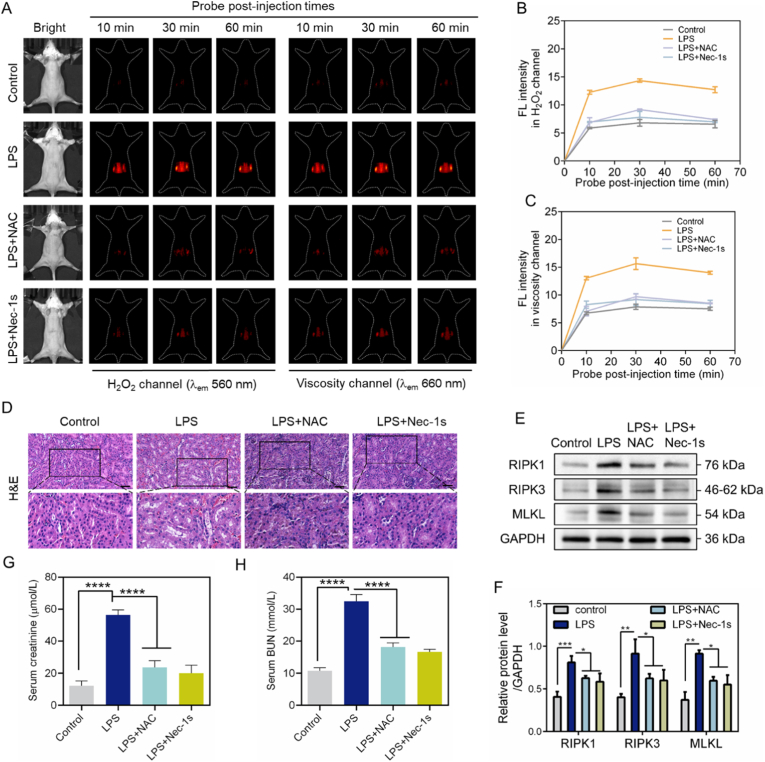
Imaging-based monitoring of therapeutic modulation of redox-biophysical remodeling in LPS-induced AKI. (A) Fluorescence images of living mice injected with PB-PB-B(OH)2 after different treatments. NAC (400 mg/kg) or Nec-1s (1.5 mg/kg) was administered 30 min before LPS challenge. (B–C) The dynamic fluorescence intensity of kidneys as a function of time post-injection of probe in living mice after LPS treatment for 24 h, then with or without NAC or Nec-1s, respectively. (D) Representative H&E staining of kidney tissue sections. Scale bar: 50 μm. (E) Western blotting analysis of necroptosis-related proteins RIPK1, RIPK3, and MLKL. (F) Quantification analysis of RIPK1, RIPK3, and MLKL normalized to GAPDH in (E). (G-H) Quantification of renal injury severity *via* Cr (G), and BUN (H). Data are shown as mean ± SD (n = 5); statistical significance was determined by one-way ANOVA with Tukey's multiple comparison test. The compared groups are indicated by horizontal brackets in the graphs. *P < 0.05; **P < 0.01; ***P < 0.001; ****P < 0.0001.

To investigate the applicability of PB-PB-B(OH)2 in evaluating therapeutic interventions for AKI, NAC and Nec-1s were applied in an LPS-induced AKI mouse model to modulate oxidative stress and RIPK1/necroptosis-related signaling, respectively. As shown in Fig. 6A, the dual-channel fluorescence signal of LPS-treated mice was significantly enhanced, while that in the NAC or Nec-1s treatment groups decreased substantially. Quantitative analysis of fluorescence signals revealed that both H_2_O_2_ and viscosity decreased to 63% and 68% after NAC intervention, and to 54% and 69% after Nec-1s intervention, respectively (Fig. 6B and C). This suggested that both ROS scavenging and inhibition of RIPK1/necroptosis-related signaling can attenuate injury-associated redox-biophysical stress in this model. The H&E results also showed marked improvement in tissue morphology after NAC and Nec-1s treatments (Fig. 6D). As shown in Fig. 6E and F, the expression levels of necroptosis-related proteins were reduced in the NAC and Nec-1s-treated groups. This trend was consistent with changes in Cr and BUN in the drug intervention groups (Fig. 6G and H). These results indicate that NAC and Nec-1s alleviated LPS-induced renal tubular injury and reduced necroptosis-related signaling in this model. The concomitant decreases in the green and red fluorescence signals further support that PB-PB-B(OH)2 can be used for imaging-based monitoring of therapeutic modulation of oxidative stress-associated H_2_O_2_ accumulation and viscosity-related microenvironmental remodeling in LPS-induced AKI.

## 4 Conclusion

In summary, we developed PB-PB-B(OH)2 as a mitochondria-targeted dual-responsive fluorescent probe for single-excitation dual-channel imaging of H_2_O_2_-associated oxidative stress and viscosity-related microenvironmental remodeling. The probe showed distinct green-channel activation toward H_2_O_2_ and red-channel enhancement in response to increased viscosity, with limited cross-interference under the tested conditions. In TNF-α- and LPS-stimulated HK-2 cells, PB-PB-B(OH)2 visualized concurrent mitochondrial redox and viscosity changes associated with inflammatory tubular cell injury and necroptosis-related signaling. In LPS-induced AKI mice, the dual-channel fluorescence signals changed dynamically with injury progression and were attenuated by NAC or Nec-1s treatment, consistent with histological injury, renal function markers, and RIPK1/RIPK3/MLKL-related protein expression. Overall, this work establishes PB-PB-B(OH)2 as a dual-parameter molecular imaging tool for interrogating mitochondrial redox-biophysical remodeling in LPS-induced AKI and provides a preclinical imaging strategy for monitoring renal injury progression and therapeutic response.

## Author disclosures

The authors have no relationships relevant to the contents of this paper to disclose.

## CRediT authorship contribution statement

**Junjie Wang:** Conceptualization, Data curation, Funding acquisition, Investigation, Methodology, Supervision, Visualization, Writing – original draft, Writing – review & editing. **Rong Yu:** Data curation, Investigation. **Wei Chen:** Data curation. **Xiaomin Ma:** Data curation, Software. **Xingzhou Peng:** Writing – review & editing. **Fabiao Yu:** Writing – review & editing. **Yongjun Zhu:** Writing – review & editing.

## Declaration of competing interest

The authors declare that they have no known competing financial interests or personal relationships that could have appeared to influence the work reported in this paper.

## Data Availability

Data will be made available on request.
