# Supplementary material for: A mitochondria-targeted H2O2/viscosity dual-responsive fluorescent probe for visualizing redox-biophysical remodeling in LPS-induced acute kidney injury
Source: Redox Biol. 2026 Jul 10;95:104294. doi: 10.1016/j.redox.2026.104294 (PMC13377442; doi:10.1016/j.redox.2026.104294)
Supplement: Multimedia component 1 [file mmc1.docx]

**Supporting Information**

**A Mitochondria-targeted H_2_O_2_/viscosity Dual-Responsive Fluorescent Probe for Visualizing Redox-biophysical Remodeling in LPS-Induced Acute Kidney Injury**

Junjie Wang ^1, [a]^, Rong Yu ^1,[b]^, Wei Chen^[c]^, Xiaomin Ma^[c]^, Xingzhou Peng^[c]*^, Fabiao Yu^[a]^*, Yongjun Zhu^[b]^*

[a] NHC Key Laboratory of Tropical Disease Control, School of Life Science and Medical Technology, Hainan Medical University, Haikou 571199, China

[b] Department of Nephrology, Key Laboratory of Emergency and Trauma, Ministry of Education, Key Laboratory of Haikou Trauma, Key Laboratory of Hainan Trauma and Disaster Rescue, The First Affiliated Hospital, Hainan Medical University, Haikou 571199, China

[c] School of Biomedical Engineering, Hainan University, Haikou 570228, China

^1^ These two authors contributed equally to this work (J. Wang and R. Yu)

*Correspondence: Xingzhou Peng (pengxzh@hainanu.edu.cn)

Fabiao Yu (yufabiao@muhn.edu.cn)

Yongjun Zhu (zyj418207195@yeah.net)

Table of Contents

General experimental section

Synthesis of compound PB-PB-B(OH)2

^1^H, ^13^C NMR, and HRMS spectra (Figures S1-S4)

Photophysical characterization and imaging data (Figures S5–S21)

Comparison of reported dual-responsive probes (Table S1)

Photophysical properties of PB-PB-B(OH)2 (Table S2)

Viscosities of glycerol/PBS mixtures (Table S3)

**General Experimental Section**

**Materials and instruments:** All chemicals were purchased from commercial suppliers and used without further purification. All chemicals used in the synthesis were of analytical grade.

Absorption spectra were measured using a Shimadzu UV-2700 spectrophotometer. Fluorescence spectra were recorded using a Horiba Fluorolog-QM fluorescence spectrophotometer. ^1^H NMR and ^13^C NMR spectra were recorded with a Bruker Ascend^TM^ 400 Ultrashield spectrometer with tetramethylsilane (TMS) as an internal standard. High-resolution mass spectra (HRMS) were measured on a Bruker ESI-Q-TOF-MS/MS mass spectrometer. Confocal fluorescence images were acquired using an Olympus FV-3000 confocal laser scanning microscope. Two-photon fluorescence images were recorded on Olympus FVMPE-RS microscopy imaging system. Cytotoxicity of PB-PB-B(OH)2 was evaluated using a standard CCK-8 assay on a SpectraMax i5 microplate reader. Flow cytometer analysis was performed using a BD Accuri C6 Plus flow cytometer. *In vivo* imaging experiments were conducted on a PerkinElmer IVIS Lumina XRMS Series III imaging system.

Measurements of the two-photon absorption cross-section: Two-photon absorption cross-section (*δ*) describes the absorption ability of a fluorophore under two-photon excitation. The *δ* value of PB-PB-B(OH)2 was measured *via* a two-photon-induced fluorescence method. ^[1]^

The *δ* values over 800-1000 nm were measured based on a commercial upright two-photon fluorescence scanning microscope (Olympus, water-immersed objective, 25×/1.00), equipped with a Ti:sapphire fs laser. The fs laser beam was focused on a capillary (containing PB-PB-B(OH)2 in the PBS buffer) by the objective, and the two-photon excited fluorescence signals were collected by the same objective. Rhodamine 6G (Rh6G) in methanol was used as reference. ^[2]^

| $\frac{\delta_{1}}{\delta_{2}}=\frac{F_{1}\phi_{2}c_{2}n_{2}}{F_{2}\phi_{1}c_{1}n_{1}}$ |
| --- |

In this equation, *δ* stands for the two-photon absorption cross-section, *F* is the two-photon fluorescence intensity, $\phi$ is the fluorescence quantum yield, *c* is the molar concentration, *n* is the refractive index of the solution, and the subscripts 1 and 2 represent the sample (PB-PB-B(OH)2 in PBS buffer) and the reference (Rh6G in methanol), respectively. The fluorescence quantum yield of PB-PB-B(OH)2 in PBS was determined to be 0.19 by the relative method. ^[3]^ As the parameters of Rh6G are available, the *δ* of PB-PB-B(OH)2 at 800-1000 nm can be calculated accordingly.

Optical spectral measurements: A stock solution of PB-PB-B(OH)2 was prepared in DMSO at 1 mM and stored at 4 °C in the dark. The probe was prepared by diluting the probe stock solution into the PBS solution (10 μM). The fluorescence excitation wavelength was 410 nm, and the emission was collected at 430-820 nm or 550-800 nm. For the H_2_O_2_ response assay, the test solution was prepared by adding the probe stock solution, different concentrations of H_2_O_2_ were added to give a final volume of 2 mL. For the viscosity response assay, the probe stock solution was added to glycerol/PBS mixture to give a final volume 2 mL. For selectivity experiments, the probe was incubated with H_2_O_2_ or other analytes, such as reactive oxygen species, amino acids, cations, and anions for 30 min. The limit of detection (LOD) and limit of quantification (LOQ) for H_2_O_2_ were calculated according to LOD = 3σ/*k* and LOQ = 10σ/*k*, where σ represents the standard deviation of blank measurements and *k* represents the slope of the calibration curve obtained from the linear relationship between fluorescence intensity at 547 nm and H_2_O_2_ concentration.

Apoptosis/necroptosis assessment: An Annexin V-FITC/PI apoptosis detection kit was used to assess apoptotic and necrotic cell death. Briefly, a 6-well plate (1 $\times$ 10^5^ cells/well) was used to culture HK-2 cells. HK-2 cells were stimulated with TNF-$\alpha$ (100 ng/mL) for 6, 12, and 24 h. To inhibit ROS production, HK-2 cells were pretreated with NAC (1 mM) or Nec-1s (1 μM) and then stimulated with TNF-$\alpha$ for 24 h. All cells were then collected and stained with 5 μL Annexin V-FITC and 5 μL PI, followed by immediate flow cytometry analysis, and the data were analyzed using FlowJo software.

Western blotting: HK-2 cells and kidney tissue were lysed in RIPA buffer containing protease inhibitors. Protein concentrations were determined using a BCA assay (Beyotime, China). Proteins were separated by 10% SDS-PAGE and transferred to polyvinylidene difluoride membranes (Millipore, USA). Membranes were blocked with 5% non-fat milk in Tris-buffered saline with Tween-20 (TBST; Solarbio, Beijing) for 1 h at room temperature, then incubated overnight at 4 °C with the following primary antibodies: anti-RIPK1 (1:500, ABclonal, USA), anti-RIPK3 (1:2000, Proteintech, China), anti-MLKL (1:1000, NOVUS, USA), and anti-GAPDH (1:5000, Servicebio, China). After washing, membranes were incubated with a goat anti-rabbit secondary antibody (1:2000, Proteintech, China) for 1 h. Protein blots were visualized and analyzed using an integrated chemiluminescent gel imaging system (Sinsage, China).

**Synthesis of compound 1:** A solution of 4-methylpyridine (1 eq.) and 4-(bromomethyl)phenylboronic acid (1 eq.) in CH_3_CN (100 mL) was stirred at 80-90 °C for 16 h. The solvent was evaporated under vacuum to afford compound **1** as a yellow oil, which was used for the next step without further purification. ^1^H NMR (400 MHz, DMSO-*d*6) $\delta$ (ppm) 9.02 (s, 2H), 8.00 (d, *J* = 6.0 Hz, 2H), 7.82 (d, *J* = 7.8 Hz, 1H), 7.54 – 7.38 (m, 4H), 7.38 – 7.10 (m, 1H), 5.77 (s, 2H), 2.60 (s, 3H).

**Synthesis of PB-PB-B(OH)2:** A mixture of aldehyde (1 eq.), compound **1** (1.05 eq.) and piperidine (1 drop) in EtOH was stirred at 80 °C for 16 h. The solvent was evaporated under vacuum. The residue was purified by recrystallization from EtOAc/EtOH to afford the target compounds as a dark brown solid. ^1^H NMR (400 MHz, CDCl_3_) δ 8.90 (s, 2H), 7.68 (s, 2H), 7.44 (d, J = 19.2 Hz, 4H), 7.40 (s, 2H), 7.30 (s, 3H), 6.79 (d, J = 7.6 Hz, 2H), 6.73 (d, J = 16.0 Hz, 1H), 5.80 (s, 2H), 3.29 (s, 4H), 1.60 (s, 7H). ^13^C NMR (101 MHz, DMSO) δ 154.52 (s), 153.01 (s), 144.02 (2C), 142.68 (s), 135.14 (s), 130.79 (2C), 129.71 (2C), 129.68(s), 128.95 (2C), 124.16 (s), 123.36 (2C), 118.28 (s), 114.66 (2C), 62.34 (s), 48.42 (2C), 25.37 (2C), 24.40 (s). HRMS: m/z calcd for C_25_H_30_BBrN_2_O_2_^+^: [M]^+^ 478.1427; found: 478.1422.


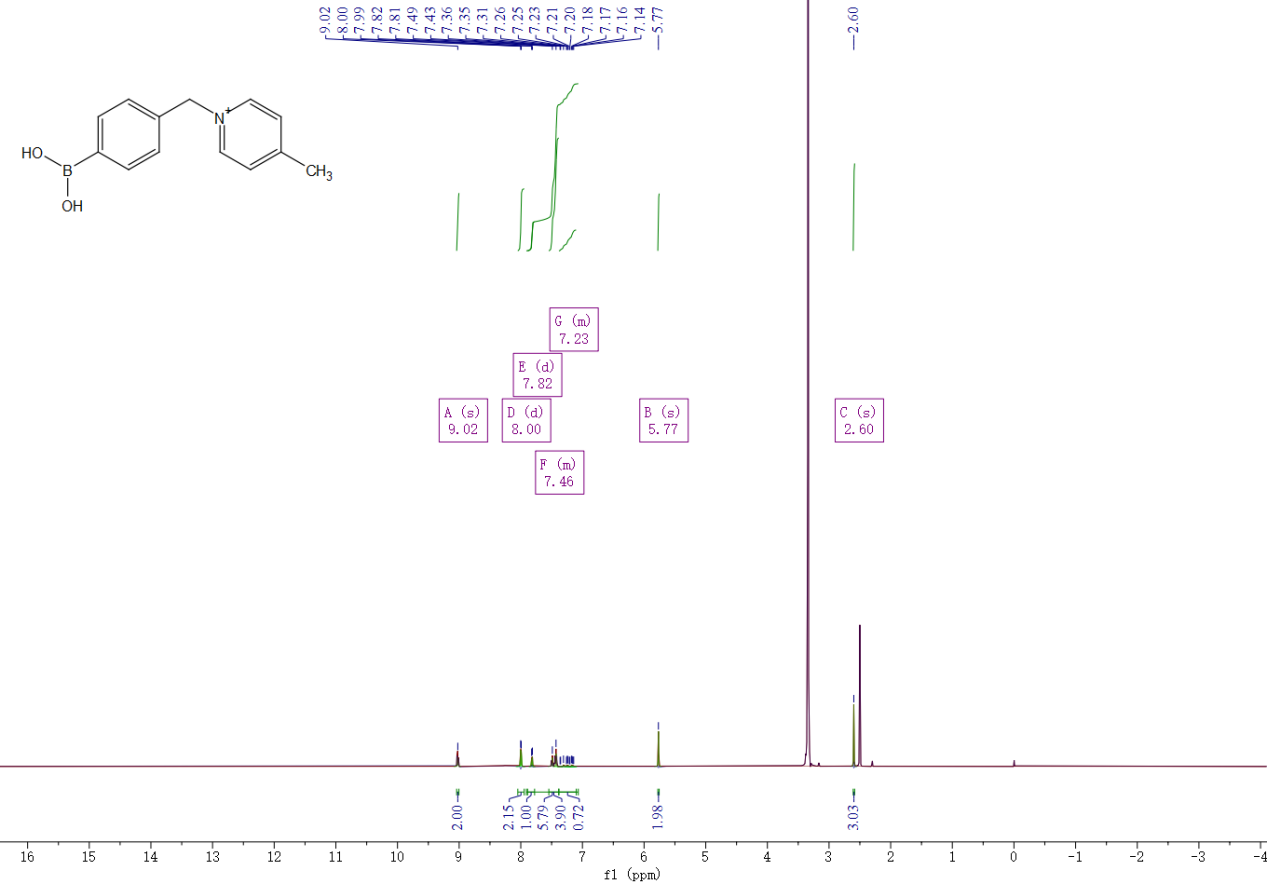


Figure S1. ^1^H NMR spectrum of compound **1**.


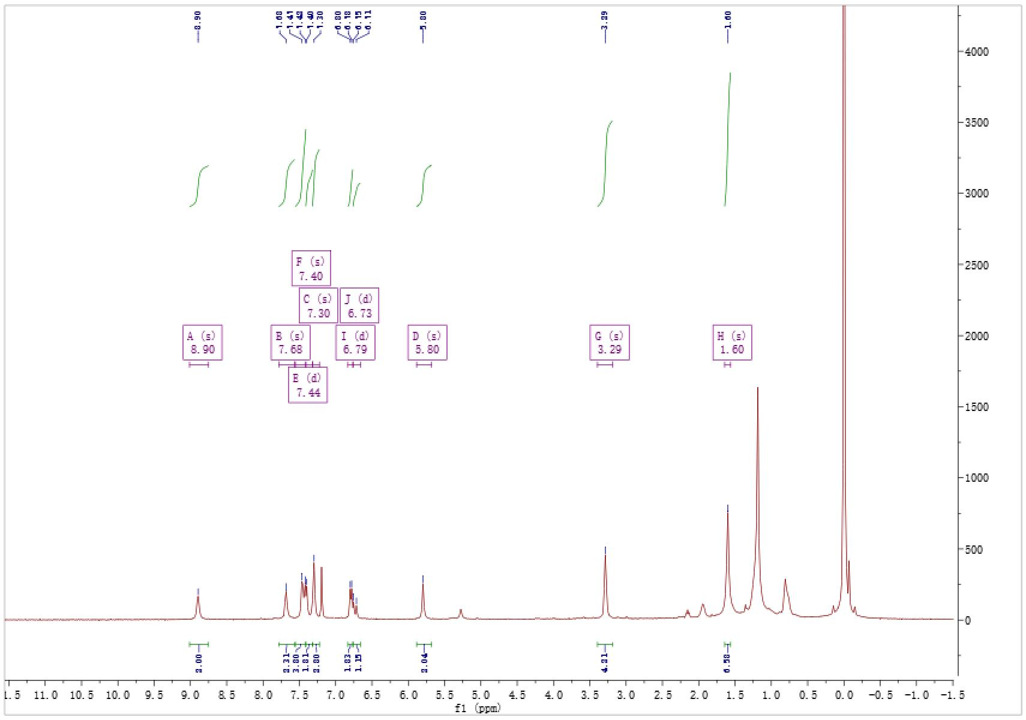


Figure S2. ^1^H NMR spectrum of PB-PB-B(OH)2.


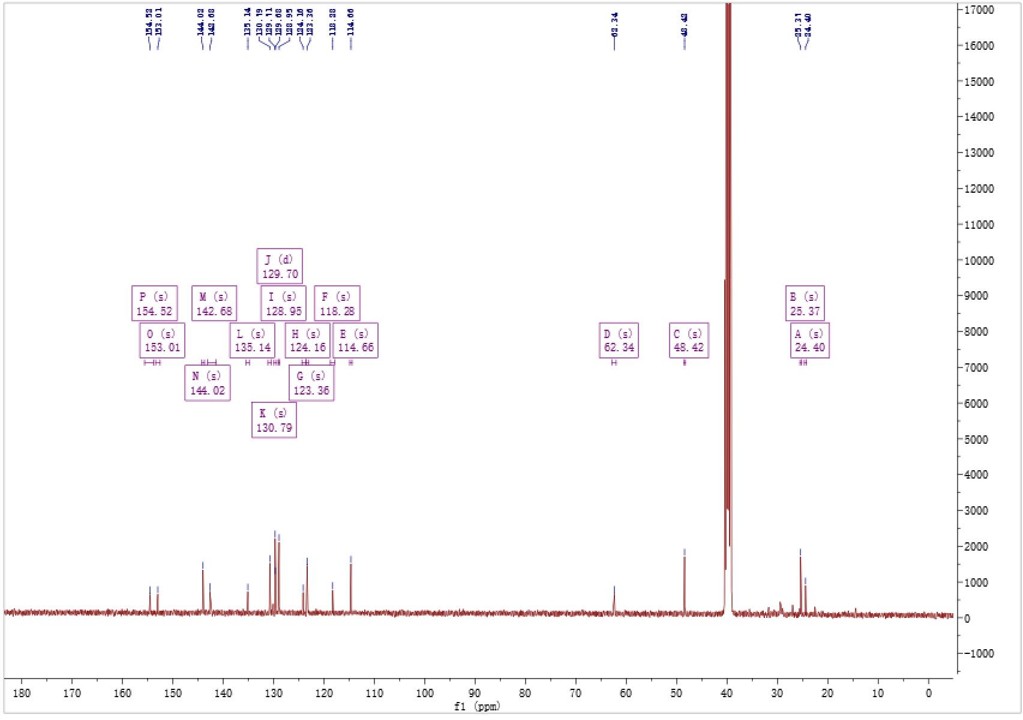


Figure S3. ^13^C NMR spectrum of PB-PB-B(OH)2.


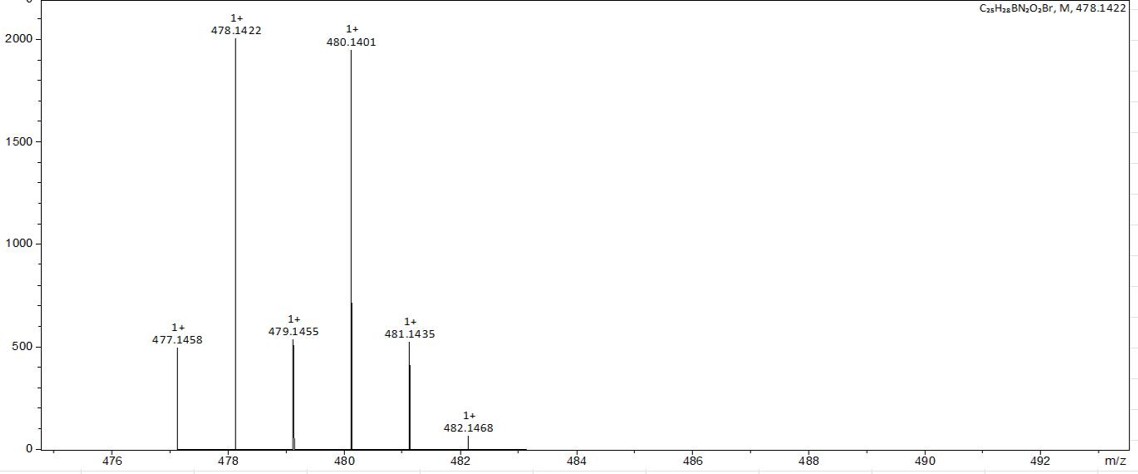


Figure S4. Mass spectrum of PB-PB-B(OH)2.

Table S1. Comparison of dual-responsive fluorescent probes for ROS and viscosity.

| Representative probe | Key feature | Main model/application | References |
| --- | --- | --- | --- |
| PB-PB-B(OH)2 | Two-photon dual-imaging of mitochondrial H_2_O_2_ and viscosity for visualization of necroptosis-associated tubular injury | AKI mouse models | This work |
| HX-VP-2 | NIR dual-imaging of mitochondrial ONOO^-^ and viscosity | NAFLD mouse models | *Sens. Actuators B Chem.*, **2025**, 433, 137580. |
| YLS | NIR dual-response fluorescent probes for ONOO^-^ and viscosity | Rheumatoid arthritis (RA) mouse models | *Chin. Chem. Lett.*, **2025**, 36, 110713. |
| Mito-IQS | Mitochondria-targeted fluorescence probe viscosity and H_2_O_2_ | Cerebral ischemia–reperfusion injury (CIRI) mouse models | *Anal. Chem.*, **2024**, 96, 3436–3444. |
| AS-CN | NIR dual-imaging of mitochondrial HOCl and viscosity | Atherosclerosis mice models | *Chem. Eng. J.*, **2023**, 464, 142687. |
| NS | Mitochondria-targeted fluorescence probe for viscosity and HClO | Atherosclerosis-related foam cells | *Spectrochim. Acta A Mol. Biomol. Spectrosc.,* **2023**, 303, 123225. |
| P-HV | BODIPY-based dual-imaging of H_2_O_2_ and viscosity for visualization of palmitic acid (PA)-induced pyroptosis | Cells and mice | *Chem. Commun.,* **2023**, 59, 12775–12778. |
| CQ | dual-response fluorescent probes for simultaneous detection of ONOO^-^ and viscosity | NAFLD models | *Anal. Chem.*, **2022**, 94, 17439–17447. |
| Mito-ONOO | Two-photon ratiometric dual-response fluorescent probes for ONOO^-^ and viscosity | Cellular and mouse stroke models | *Chem. Sci.*, **2022**, 13, 5363–5373. |
| ADM | Dual-response fluorescent probe for viscosity and H_2_O_2_ | Cellular inflammation imaging | *Talanta,* **2021**, 235, 122719 |
| TPP-Tba | Mitochondria-targeted AIE-active dual-imaging for H_2_O_2_ and viscosity | Cell and tissue for fatty liver | *New J. Chem.,* **2021**, 45, 12138–12144. |

Table S2. Summary of photophysical properties of PB-PB-B(OH)2 in PBS buffer.

|  | $\lambda_{max}^{abs}$[a] | $\lambda_{max}^{em}$[b] | $\phi_{FL}$[c] | $\delta_{max}$[d] |
| --- | --- | --- | --- | --- |
| PB-PB-B(OH)2 | 410 | 547 | 0.19±0.015 | 71.6 |

[a] Peak position of the absorption band. [b] Peak position of the emission, excited at the absorption maximum in PBS. [c] Fluorescence quantum yield. [d] The maximum two-photon absorption cross-section values upon excitation at 850 nm in GM (1 GM = 10^-50^ cm^4^ s photon^-1^).

Table S3. The viscosities of glycerol/PBS mixture at different volume proportions.

| Glycerol (V%) | PBS (V%) | Viscosity (cP) | Log($\eta$) |
| --- | --- | --- | --- |
| 0 | 100 | 0.99 | -0.004 |
| 10 | 90 | 1.41 | 0.149 |
| 20 | 80 | 3.03 | 0.481 |
| 30 | 70 | 5.71 | 0.757 |
| 40 | 60 | 7.64 | 0.883 |
| 50 | 50 | 12.4 | 1.093 |
| 60 | 40 | 17.3 | 1.238 |
| 70 | 30 | 53 | 1.724 |
| 80 | 20 | 135 | 2.13 |
| 90 | 10 | 261 | 2.417 |
| 99 | 1 | 953 | 2.979 |


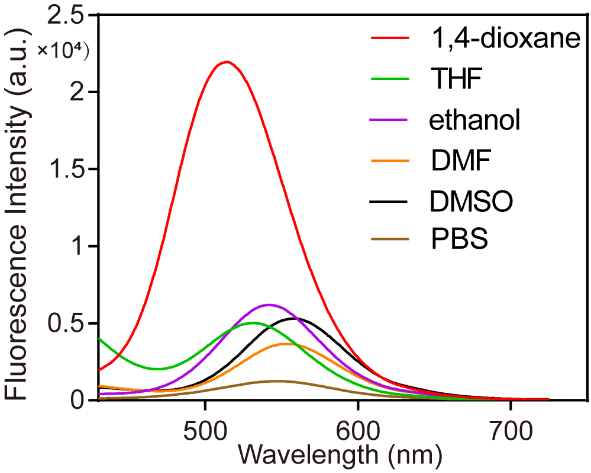


Figure S5. Fluorescence emission spectra of the probe (10 μM) in different polar solvents ($\lambda_{ex}$= 410 nm).


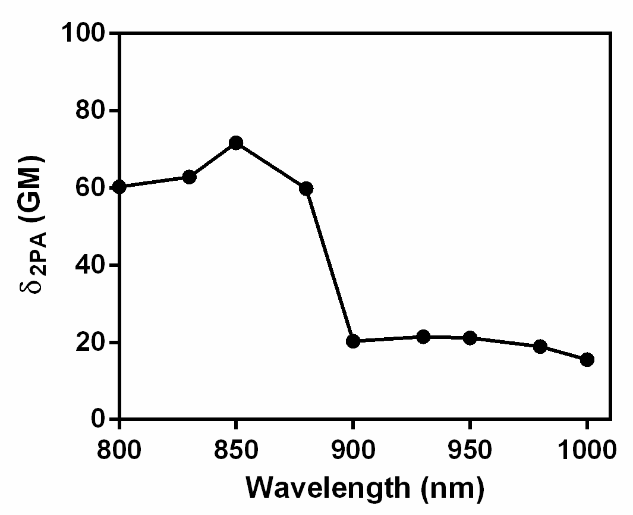


Figure S6. Two-photon absorption cross-section of PB-PB-B(OH)2 at different wavelengths.





Figure S7. Fluorescence intensities of probe at 663 nm in PBS-glycerol (0-100%).


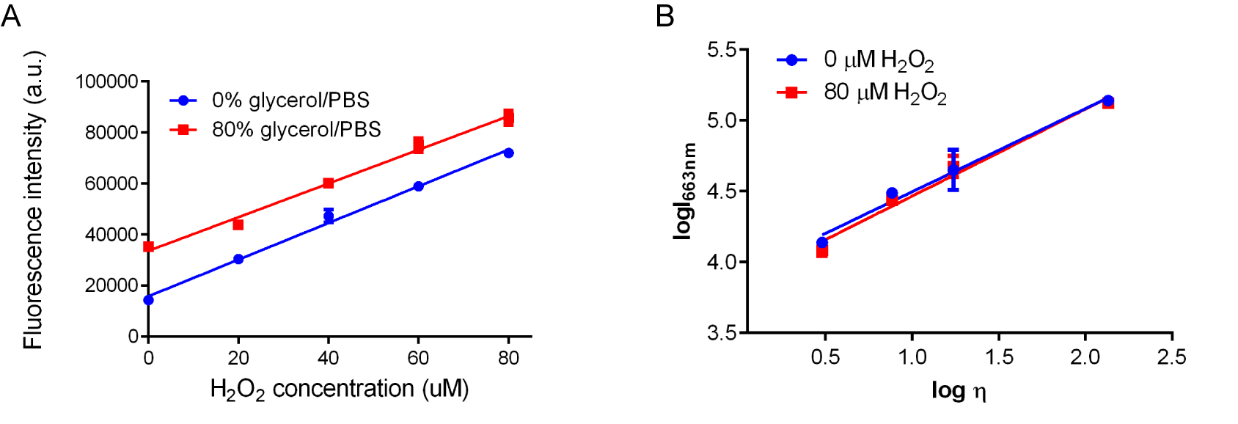


Figure S8. Cross-interference evaluation between H_2_O_2_ and viscosity responses of PB-PB-B(OH)2. (A) Linear relationship between fluorescence intensity and H_2_O_2_ concentrations in PBS or 80% glycerol/PBS. (B) Linear relationship of fluorescence intensity (I₆₆₃) and viscosity (η) described with Förster-Hoffmann equation in the absence or presence of H_2_O_2_.





Figure S9. Fluorescence intensity at 547 nm in the presence of different biological species. 1: Cl⁻; 2: CO₃²⁻; 3: SO₄²⁻; 4: HPO₄²⁻; 5: H_2_PO_4_^-^ ^-^; 6: SO_3_^2^; 7: NO_2_^-^; 8: S^2-^; 9: Cu^2+^; 10: Cu^+^; 11: Fe^2+^._._


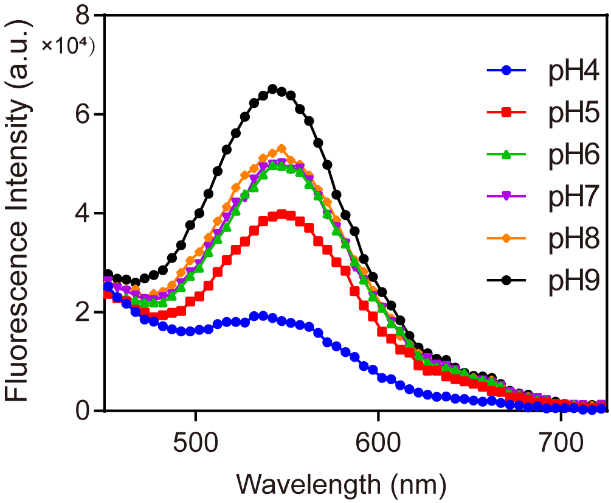


Figure S10. Fluorescence emission spectra of probe (10 μM) under different pH conditions ($\lambda_{ex}$= 410 nm).





Figure S11. Cytotoxicity of probe with different concentrations in HK-2 cells.


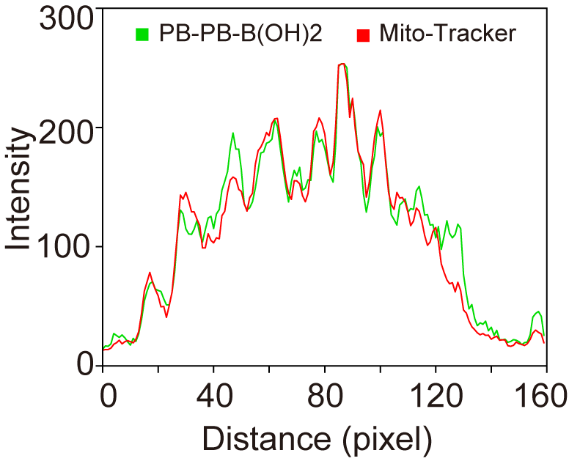


Figure S12. Intensity profiles for regions of interest of overlapped image in Figure 1A (black line)


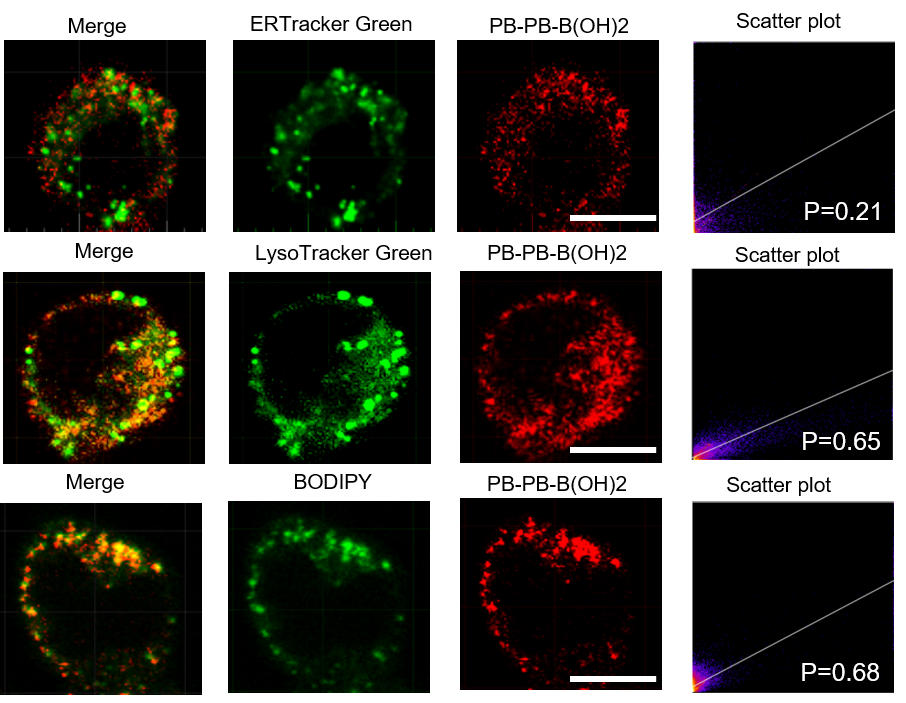


Figure S13. Subcellular colocalization of PB-PB-B(OH)2 with organelle-specific markers. Imaging parameters: PB-PB-B(OH)2 (λₑₓ = 405 nm, λₑₘ = 480-580 nm); ERTracker Green (λₑₓ = 488 nm, λₑₘ = 500-550 nm); LysoTracker Green (λₑₓ = 488 nm, λₑₘ = 500-550 nm); BODIPY 493/503 (λₑₓ = 488 nm, λₑₘ = 500-530 nm). Scale bar: 10 μm.


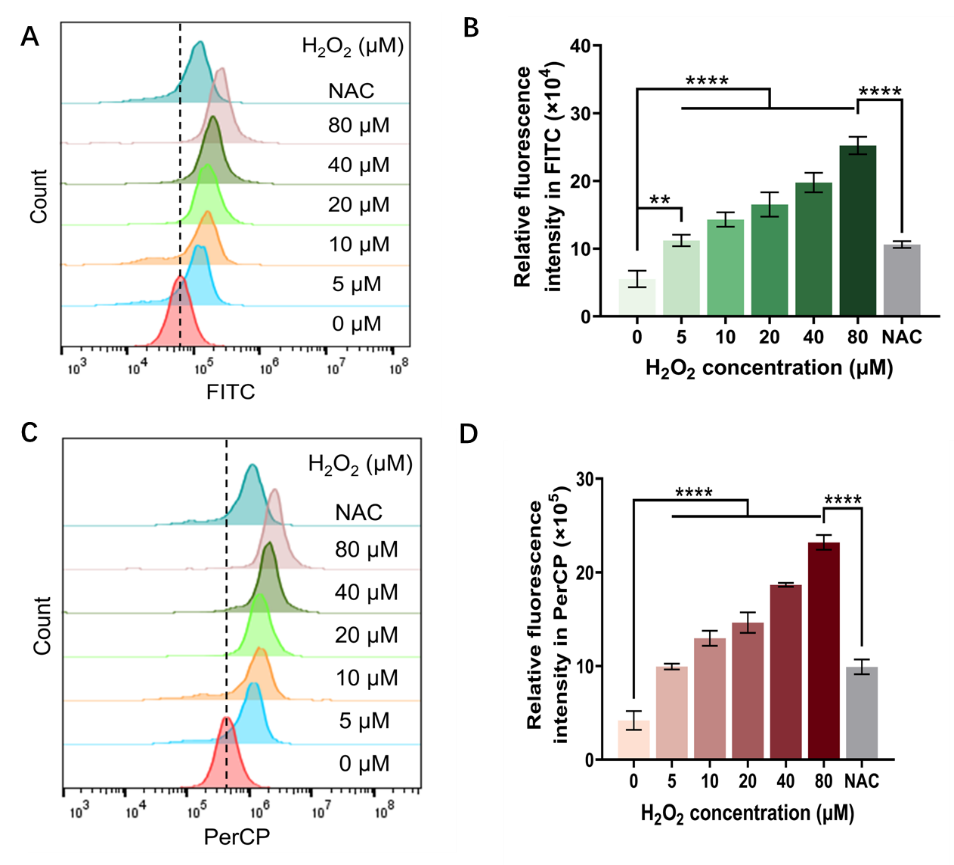


Figure S14. Flow cytometric analysis of mitochondrial H_2_O_2_ changes in HK-2 cells under different concentrations of H_2_O_2_ (0-80 μM) or NAC (1 mM) for 30 min. (A-B) H_2_O_2_ channel; (C-D) viscosity channel.


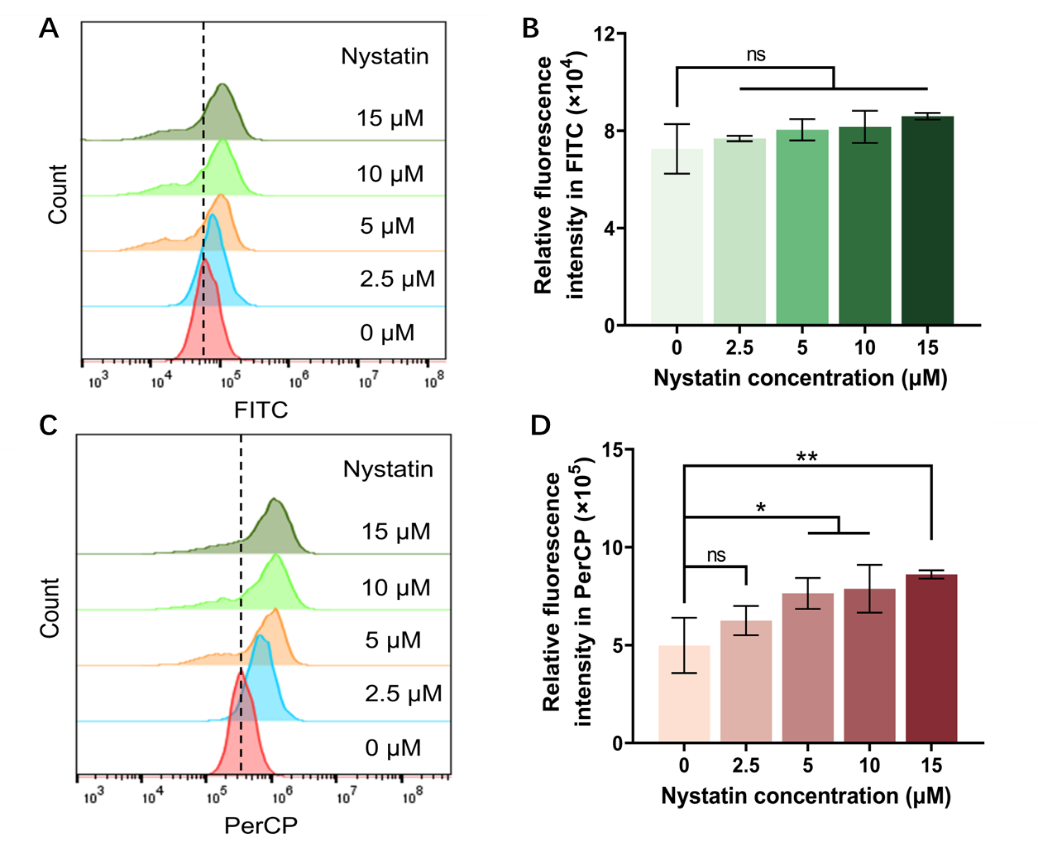


Figure S15. Flow cytometric analysis of mitochondrial viscosity changes in HK-2 cells under different concentrations of nystatin (0-15 μM) for 30 min. (A-B) H_2_O_2_ channel; (C-D) viscosity channel.







Figure S16. Quantification analysis of necroptosis-related proteins RIPK1, RIPK3, and MLKL with or without NAC and Nec-1s.







Figure S17. Quantification analysis of necroptosis relative proteins RIPK1, RIPK3, and MLKL after LPS treatment for 24 h, with NAC and Nec-1s.


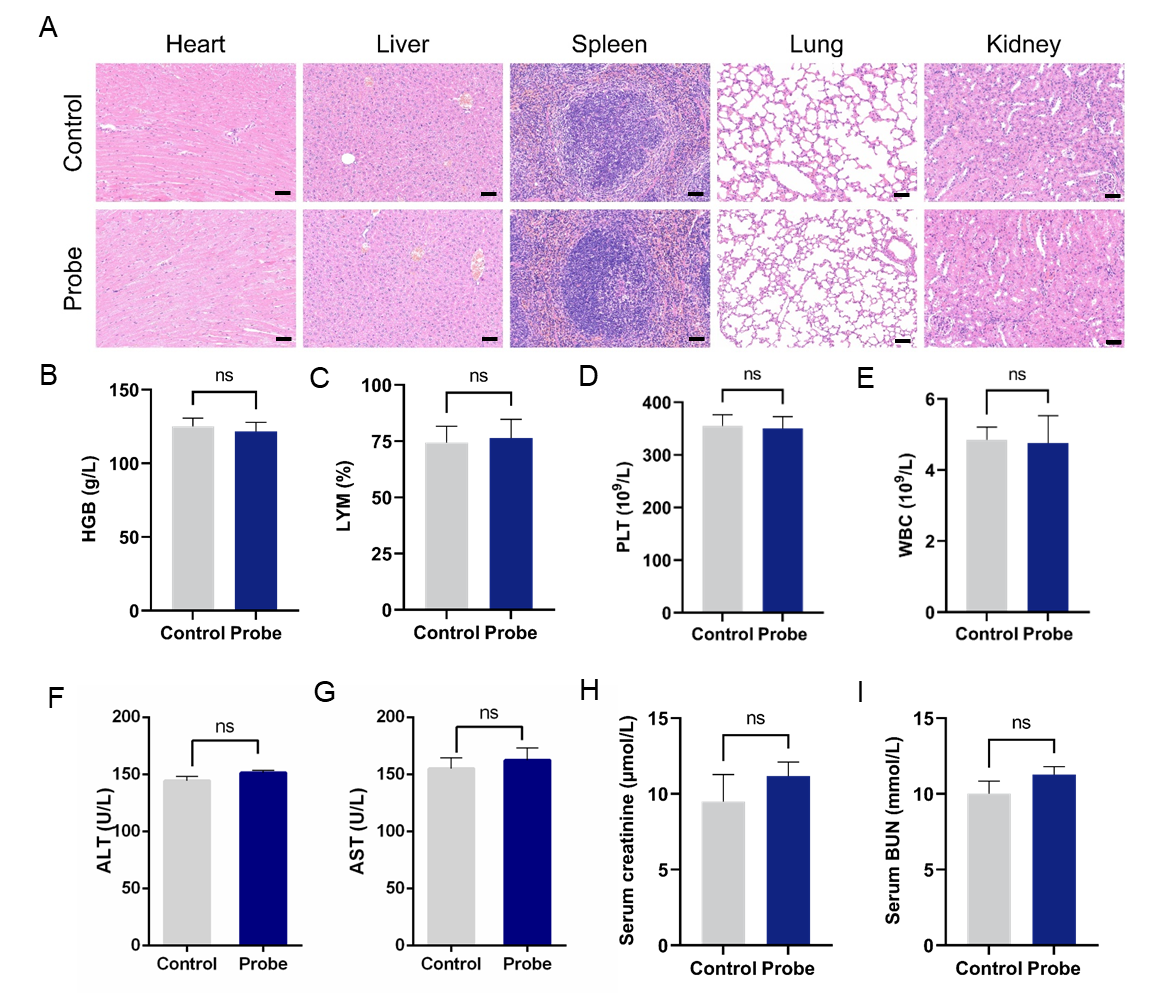


Figure S18. *In vivo* biocompatibility assessment of PB-PB-B(OH)2 probe. (A) Representative H&E-stained histological sections of major organs (heart, liver, spleen, lung, kidney) 24 h after probe injection in AKI mice. (B–E) Levels of hemoglobin concentration (HGB), lymphocytes (LYM), platelets (PLT) and white blood cells (WBC). (F-I) Blood biochemical analysis of liver and kidney function. ALT: alanine aminotransferase; AST: aspartate aminotransferase; Cr: creatinine; BUN: blood urea nitrogen. ns, not statistically significant compared to the control (PBS) group (*p* > 0.05).


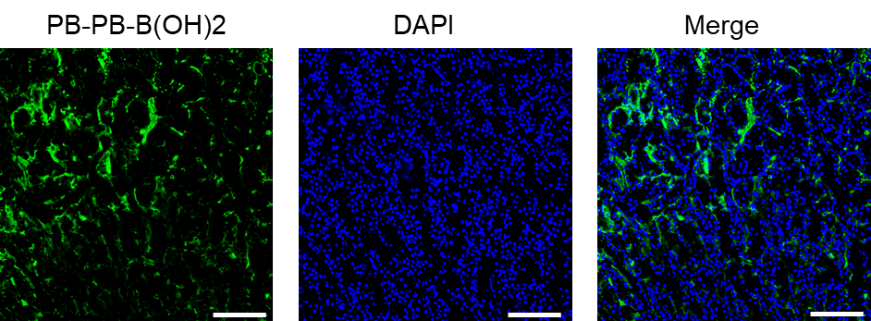


Figure S19. Representative confocal fluorescence microscopy images of kidney slices from mice with *i.v.* injection of PB-PB-B(OH)2 at 24 h post-treatment with LPS. Scale bar: 100 μm.


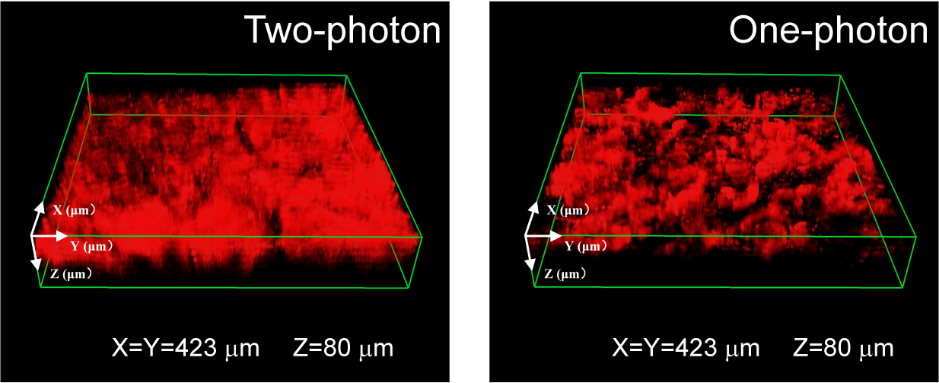


Figure S20. Comparison of two-photon and one-photon fluorescence imaging of kidney tissue.





Figure S21. Western blotting analysis of necroptosis relative proteins RIPK1, RIPK3, and MLKL after treatment with LPS (10 mg/kg) for 12, 24 and 48 h. Quantification analysis of RIPK1, RIPK3, and MLKL normalized to GAPDH. Significant differences were analyzed by one-way ANOVA with **P* < 0.05; ** *P* < 0.01; *** *P* < 0.001; **** *P* < 0.0001.

Reference:

1. Y. Wang, R. Hu, W. Xi, F. Cai, S. Wang, Z. Zhu, R. Bai and J. Qian, *Biomed. Opt. Express*, 2015, **6**, 3783.

2. D. A. Oulianov, I. V. Tomov, A. S. Dvornikov and P. M. Rentzepis, *Opt. Commun.*, 2001, **191**, 235.

3. A. T. R. Williams, S. A. Winfield and J. N. Miller, *Analyst*, 1983, **108**, 1067.
